# Optogenetic‐Controlled iPSC‐Based Vaccines for Prophylactic and Therapeutic Tumor Suppression in Mice

**DOI:** 10.1002/advs.202416115

**Published:** 2025-07-06

**Authors:** Longliang Qiao, Lingxue Niu, Zhihao Wang, Di Dai, Shasha Tang, Xiaoding Ma, Zhenqiang Deng, Guiling Yu, Yang Zhou, Tao Yan, Xingwan Liu, Deqiang Kong, Linfeng Hu, Xiang Li, Junwei Zhao, Fengfeng Cai, Meiyan Wang, Haifeng Ye

**Affiliations:** ^1^ Shanghai Frontiers Science Center of Genome Editing and Cell Therapy Biomedical Synthetic Biology Research Center Shanghai Key Laboratory of Regulatory Biology Institute of Biomedical Sciences and School of Life Sciences East China Normal University Dongchuan Road 500 Shanghai 200241 China; ^2^ Department of Breast Surgery Tongji Hospital School of Medicine Tongji University Xincun Road 389 Shanghai 200065 China; ^3^ Wuhu Hospital Health Science Center East China Normal University Middle Jiuhua Road 263 Wuhu China; ^4^ Beijing Life Science Academy Beijing 102209 China; ^5^ School of Medicine Shanghai University Shanghai 200444 China; ^6^ Chongqing Key Laboratory of Precision Optics Chongqing Institute of East China Normal University Chongqing 401120 China

**Keywords:** immune adjuvants, iPSC, optogenetic, tumor vaccine

## Abstract

Induced pluripotent stem cells (iPSCs) share similar cellular features and various antigens profiles with cancer cells. Leveraging these characteristics, iPSCs hold great promise for developing wide‐spectrum vaccines against cancers. In practice, iPSCs are typically combined with immune adjuvants to enhance antitumor immune responses; however, traditional adjuvants lack controllability and can induce systemic toxicity, which has limited their broad application. Here, a red/far‐red light‐controlled iPSC‐based vaccine (RIVA) based on the chimeric photosensory protein FnBphP and its interaction partner LDB3 is developed; RIVA preserves the intrinsic tumor antigens of iPSCs and enables optogenetic control of an immune adjuvant's (IFN‐β) expression under red light illumination. Experiments in multiple mouse tumor models demonstrate that RIVA inhibits tumor growth and improves animal survival in prophylactic and therapeutic settings, including against pulmonary metastatic 4T1 breast cancer. RIVA efficiently stimulates dendritic cell maturation, eliciting innate immune activation effects through NK cells and elicit adaptive immune anti‐tumor responses through CD4^+^ and CD8^+^ T cells. Moreover, RIVA protects animals against tumor re‐challenge by inducing strong immunological memory, with minimal systemic toxicity. This study demonstrates RIVA as an effective optogenetic approach for developing safe multi‐antigen vaccines for the prevention and treatment of cancer.

## Graphical abstract

This study develops an a red/far‐red light‐controlled iPSC‐based vaccines (RIVA) based on the chimeric photosensory protein FnBphP and its interaction partner LDB3 for cancer immunotherapy. The RIVA preserves the multiple intrinsic tumor antigens of iPSCs and enables optogenetic control of the expression of the immune adjuvant type I interferon β under red light illumination.

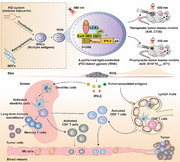

## 1 Introduction

The term “tumor vaccine” describes an immune intervention strategy that uses tumor‐specific antigens or tumor‐associated antigens and adjuvants to stimulate the antigen presentation process of dendritic cells (DCs) to initiate antitumor T‐cell immunity for cancer immunotherapy.^[^ ^1^, ^2^, ^3^, ^4^, ^5^ ^]^ The choice of antigen is typically conceptualized as the most important component of cancer vaccine design. Previous studies have demonstrated that multiple antigen vaccines are significantly more effective than single antigen vaccines in inhibiting the progression and growth of cancers.^[^ ^3^, ^6^ ^]^ Recently, induced pluripotent stem cell (iPSC)‐based cancer vaccines have been developed to view as a potential source for wide‐spectrum tumor‐related antigens to prime the immune system to target cancers, because iPSCs are known to share both tumor‐specific antigens (TSAs) and tumor associated antigens (TAAs) with cancer cells.^[^ ^6^, ^7^, ^8^, ^9^ ^]^ Work in this area has shown that iPSCs alone do not induce adequately significant anti‐tumor immune responses, so they are typically combined with an adjuvant to improve antitumor immune responses.^[^ ^7^, ^9^ ^]^ Moreover, packaging antigens and adjuvants into one entity is more efficient at inducing cytotoxic T‐cell activity compared to their free counterparts.^[^ ^10^, ^11^ ^]^

Although conventional innate immune stimuli contribute to immune activation, they could induce exhausted immune cells, resulting in suboptimal cancer immunotherapy.^[^ ^12^ ^]^ In addition, systemic administration of immunostimulatory agents is often accompanied with severe side effects which limit dosing, and thereby efficacy.^[^ ^13^, ^14^, ^15^ ^]^ Therefore, it is necessary to develop technologies for controlling the location and duration of the immune response for efficient vaccination. Previous studies have demonstrated biomaterials‐based delivery systems capable of release of immune adjuvants [*e.g*., toll‐like receptor 9 (TLR9) agonist cytidine phosphate guanosine (CpG); various cytokines] to enhance cell immunogenicity and direct downstream immune responses for therapeutic benefit.^[^ ^16^, ^17^, ^18^, ^19^ ^]^ A photoactivatable toll‐like receptor 7/8 (TLR7/8) nanoagonist (PNA) system based on a copolymer was recently reported to induce immunogenic cell death (ICD) and to trigger release of adjuvants from nanoaggregates for cancer vaccination after irradiation;^[^ ^20^ ^]^ this system resulted in inhibition of both local tumor recurrence and the growth of distant tumors. However, synthetic material‐based delivery systems often pose limitations, such as potential cytotoxicity due to negative interactions with host tissues, and they lack the ability for sustained or tunable release of therapeutic agents over time.^[^ ^21^, ^22^, ^23^ ^]^

Optogenetics‐based designer cells are now providing unprecedented opportunities for traceless, remotely controlled, precision‐guided medicine.^[^ ^24^, ^25^, ^26^, ^27^, ^28^, ^29^ ^]^ Light is an attractive inducer of gene expression compared to chemical agents, given that it is noninvasive, can be delivered for a desired duration (and at specific times), and can induce fine‐tuned responses based on illumination intensity.^[^ ^24^, ^25^, ^27^, ^30^, ^31^, ^32^ ^]^ Among the many reported optogenetic systems, red/far‐red light responsive optogenetic devices have attracted special interest, owing to their effective penetration through thick biological tissues and light‐dependent reversibility.^[^ ^25^, ^33^, ^34^, ^35^, ^36^, ^37^, ^38^ ^]^ Our recently developed red/far‐red light‐responsive photoswitch system (REDMAP), based on a minimized ΔPhyA domain, exhibits rapid ON/OFF kinetics. However, its broader application, particularly in long‐term controllable gene or cell therapies, remains constrained by the dependency on phycocyanobilin (PCB), which currently requires either exogenous supplementation or cellular engineering to introduce PCB biosynthetic enzymes.^[^ ^38^ ^]^ Of particular note, red/far‐red light‐controlled systems based on the bacteriophytochrome BphPs use biliverdin (BV), a ubiquitous endogenous molecule in mammalian cells, thus obviating the need for exogenously supplying a chromophore.^[^ ^39^, ^40^, ^41^ ^]^ However, such systems are limited by low transcriptional (<10‐fold) activation in mice.^[^ ^31^ ^]^ We developed a sensitive red light‐induced protein dimerization (RID) system based on the bacterial chimeric photosensory protein FnBphP and its interaction partner LDB3 by fusing the N‐terminal extension (NTE) of a fungal phytochrome FphA from *Aspergillus nidulans* ^[^ ^36^ ^]^ to the N‐terminus of  *Dr*BphP‐PCM (photosensory core module) from *Deinococcus radiodurans*.^[^ ^41^ ^]^

**Figure 1:**
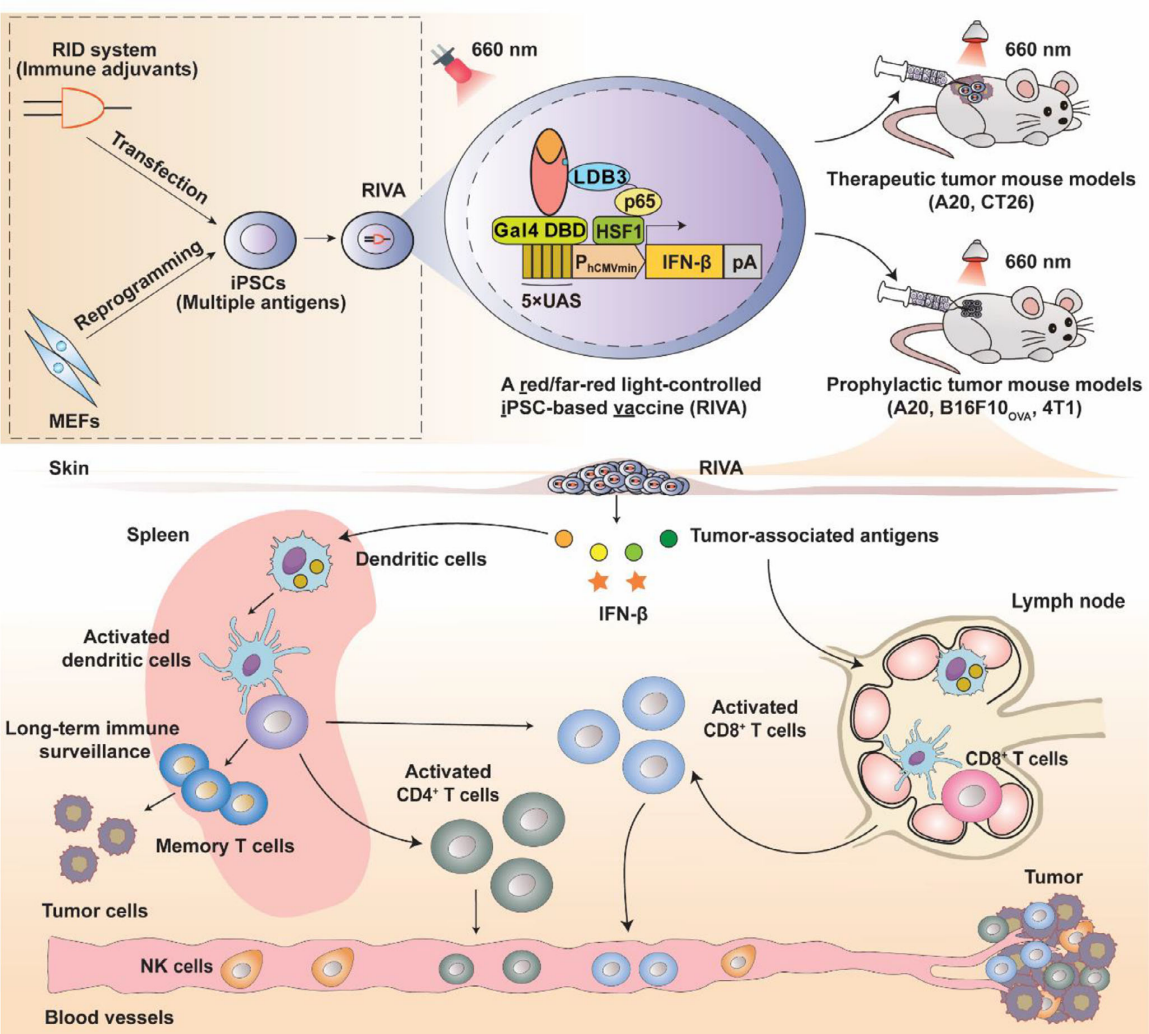
Schematic illustration of an optogenetic iPSC‐based cancer vaccine for cancer immunotherapy. A red/far‐red light‐controlled iPSC vaccine (RIVA) was developed using a red light‐induced protein dimerization (RID) system based on the chimeric photosensory protein FnBphP and its interaction partner LDB3. RIVA was generated with induced pluripotent stem cells (iPSCs) derived from mouse embryonic fibroblasts (MEFs); these cells contain multiple antigens, and the engineered cells produce and release the immune adjuvant IFN‐β when exposed to red light (RL) illumination. After treatment with mitomycin C to suppress iPSC‐derived teratomas, RIVA was implanted under the dorsum skin of mice, and efficiently stimulated dendritic cell maturation in lymph nodes and the spleen, where they can present the antigens to T cells and natural killer cells to elicit antitumor immunity to inhibit tumor growth. We investigated both therapeutic and prophylactic RIVA against multiple murine tumor models.

Using this system, we developed a red/far‐red light controlled iPSC‐based vaccine (RIVA) which combines multiple antigens derived from iPSCs with optogenetically controlled expression of one promising candidate adjuvant (type I interferon β, IFN‐β) to activate antitumor immune responses. Under red light (RL, 660 nm) illumination, FnBphP interacts with its interaction partner LDB3 to allow noninvasive control of immune adjuvant IFN‐β expression; when illuminated with far‐red light (FRL, 780 nm), FnBphP dissociates from LDB3 to terminate IFN‐β expression. We demonstrate that the optogenetic‐controlled iPSC‐based vaccines activate effective immune responses to inhibit tumor growth and resist tumor re‐challenge in multiple murine cancer models (**Figure** **1**). Our results indicate that the optogenetic‐controlled iPSC‐based vaccines mediate a safe and wide‐spectrum against tumors.

## 2 Results

### 2.1 Design and Construction of RIVA

**Figure 2:**
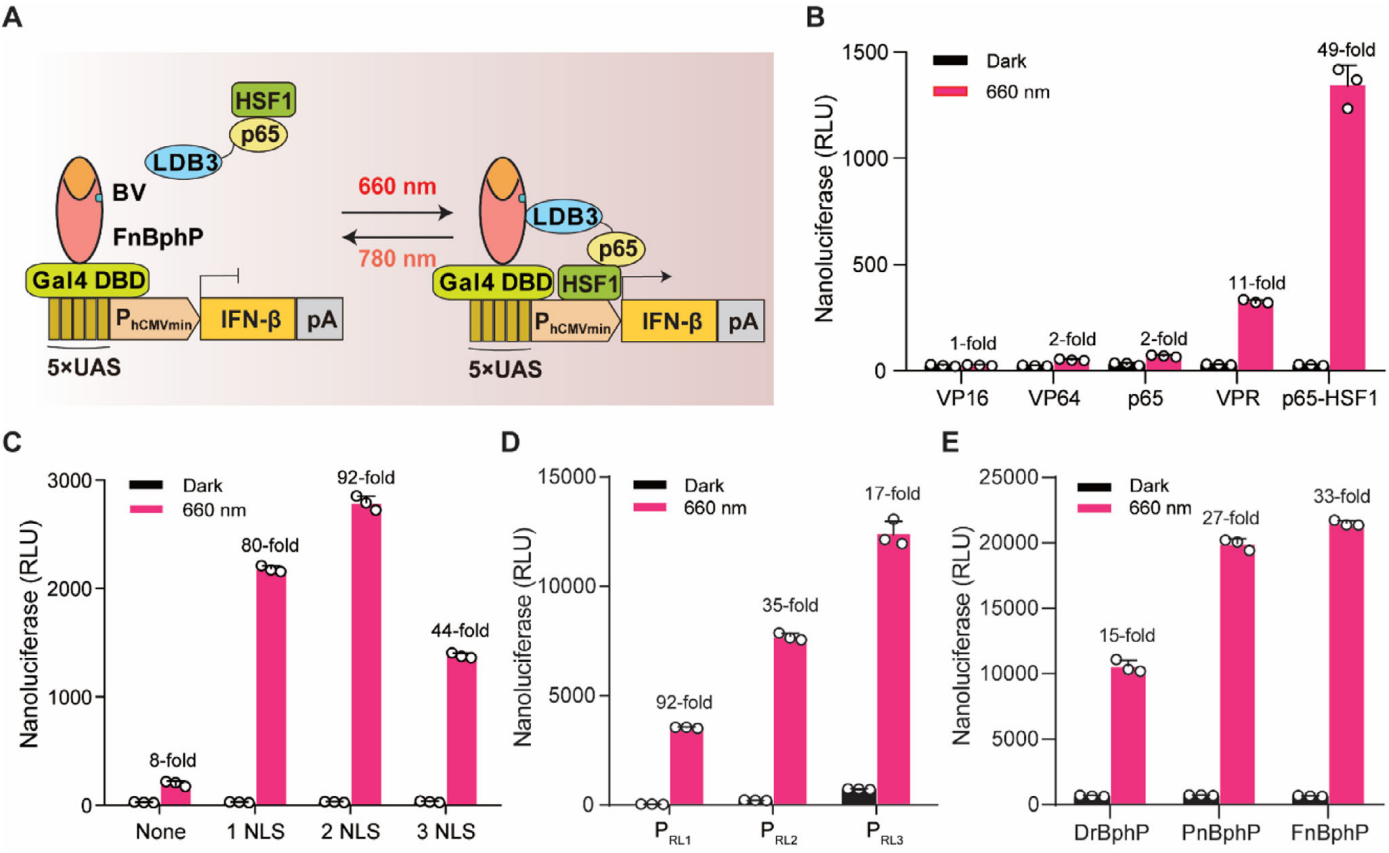
Construction and optimization of the RID system. A) Schematic showing the design of the RID system for controlling gene expression. The bacterial chimeric photosensory protein FnBphP is fused to the DNA binding domain Gal4 DBD to create a fusion light sensor domain (Gal4 DBD‐FnBphP) driven by the human cytomegalovirus promoter (P_hCMV_). Its interaction domain nanobody LDB3 is fused to the transcriptional activation domain (p65‐HSF1) to create a RL (660 nm)‐dependent transactivator (LDB3‐p65‐HSF1) driven by P_hCMV_. When exposed to RL, the transactivator (LDB3‐p65‐HSF1) can specifically bind to the light sensor domain (Gal4 DBD‐FnBphP), after which the bound complex preferentially interacts with a chimeric promoter (P_RL_, 5 × UAS‐P_hCMVmin_) and initiates transgene expression. Upon exposure to FRL (780 nm), the transactivator dissociates from the light sensor domain (Gal4 DBD‐FnBphP), leading to disengagement from the promoter and terminating transgene expression. B) Screening different transcriptional activators fused to LDB3. Prior to illumination with RL (2.0 mW cm^‐^^2^) for 24 h, iPSCs (2 × 10^4^) were co‐transfected with Gal4 DBD‐DrBphP vector (pQL217), a NanoLuc reporter expression vector (pQL259, P_RL1_‐NanoLuc‐pA; P_RL1,_ 5 × UAS‐P_TATA_‐Kozak), and one of the following expression vectors: LDB3‐VP16 (pQL232), LDB3‐VP64 (pQL234), LDB3‐p65 (pQL233), LDB3‐VPR (pQL235), or LDB3‐p65‐HSF1 (pQL236). NanoLuc production in the culture supernatant was quantified at 24 h after the initial illumination. C) Assessing how the copy number of the nuclear localization signal (NLS) of the RL‐dependent transactivator (LDB3‐p65‐HSF1) affects NanoLuc reporter expression. iPSCs (2 × 10^4^) were co‐transfected with pQL217, pQL259, and the light‐inducible transactivator with different copy numbers of NLS: 1NLS‐LDB3‐p65‐HSF1 expression vector (pNX13), 2NLS‐LDB3‐p65‐HSF1 expression vector (pNX12), 3NLS‐LDB3‐p65‐HSF1 expression vector (pQL236) or LDB3‐p65‐HSF1 expression vector (pQL256), and subsequently illuminated as described in B. NanoLuc production in the culture supernatant was quantified at 24 h after the initial illumination. D) Optimization of RL‐responsive P_RLX_‐driven NanoLuc expression. iPSCs (2 × 10^4^) were transfected with pQL217, pNX12, and a P_RLX_‐driven NanoLuc reporter [pQL259 (P_RL1_, 5 × UAS‐P_TATA_‐Kozak) or pQL264 (P_RL2_, 5 × UAS‐P_hCMVmin_) or pZW53 (P_RL3_, 5 × UAS‐P_hCMVmin_‐Kozak)], and subsequently illuminated as described in B. NanoLuc production in the culture supernatant was quantified at 24 h after the initial illumination. E) Comparison of RL inducible gene expression among the different chimeric BphP constructs fused to the Gal4 DBD domain. iPSCs (2 × 10^4^) were co‐transfected with pQL217 or Gal4 DBD‐FnBphP expression vector (pQL326) or Gal4 DBD‐PnBphP expression vector (pQL325), pNX12, and pZW53, and then illuminated with RL (2.0 mW cm^‐^^2^) for 24 h. NanoLuc production in the culture supernatant was quantified at 24 h after the initial illumination. Data in B‐E are presented as means ± SD; *n* = 3 independent experiments. Detailed descriptions of the genetic constructs and transfection mixtures are provided in Tables S1 and S2 (Supporting Information).

Seeking the capacity to modulate a controlled iPSC induced immune response while minimizing the risk of systemic toxicity, we develop a red/far‐red light‐controlled iPSC vaccine (RIVA) using the intrinsic tumor antigens of iPSCs, where iPSCs were engineered by a red light‐induced protein dimerization (RID) system based on the bacterial chimeric photosensory protein FnBphP and its interaction partner LDB3. Notably, this system does not require addition of exogenous BV as a chromophore, as BV is ubiquitous in mammalian cells and can undergo covalent bonding to FnBphP. For the RID system, the bacterial chimeric photosensory FnBphP was fused to an N‐terminal yeast Gal4 DNA binding domain (Gal4 DBD) to form a fusion light sensor (Gal4 DBD‐FnBphP). The interacting partner nanobody LDB3 was fused to p65‐HSF1 [the 65‐kDa transactivator subunit of NF‐κB (p65) and heat shock factor 1 (HSF1) transactivation domains] to form the light‐dependent transactivator LDB3‐p65‐HSF1. Upon RL (660 nm) exposure, FnBphP interacts with LDB3, enabling reconstitution of a functional complex that can bind to the P_5 × UAS_ promoter via the Gal4 DBD domain to initiate expression of the immune adjuvant IFN‐β, which has been shown as attractive candidate adjuvant for cancer vaccination.^[^ ^42^ ^]^ Conversely, upon FRL (780 nm) exposure, the FnBphP will dissociate from LDB3, which can inactivate IFN‐β expression (**Figure** **2**A).

We initially developed and selected an optogenetic transcriptional activation system by assembling *Dr*BphP fused to Gal4 DBD and LDB3 fused to five different transactivators, including VP16 (herpes simplex viral protein 16), VP64 (a tetrameric repeat of the minimal *Herpes simplex‐*derived transactivator VP16), p65 (the 65‐kDa transactivator subunit of NF‐κB), VPR (VP64‐p65‐Rta) and p65‐HSF1 [the 65‐kDa transactivator subunit of NF‐κB (p65) and heat shock factor 1 (HSF1) transactivation domains]. We transfected each of these into iPSCs derived from mouse embryonic fibroblasts (MEFs) (Figure S1, Supporting Information) and found that LDB3 fused with p65‐HSF1 resulted in the highest fold induction (49‐fold) for expression of the nanoluciferase (NanoLuc) reporter upon RL illumination compared to the dark control sample, and had relatively low background in the dark (Figure 2B).

Subsequently, we fused the LDB3‐p65‐HSF1 complex with 1 or 2 or 3 copies of a nuclear localization signal (NLS) and found that p65‐HSF1 with two copies of NLS showed the highest RL induction efficiency (92‐fold) compared to the dark control sample (Figure 2C). We also tested three RL‐responsive chimeric promoter variants (P_RL1_, 5 × UAS‐P_TATA_‐Kozak; P_RL2_, 5 × UAS‐P_hCMVmin_; P_RL3_, 5 × UAS‐P_hCMVmin_‐Kozak) for driving NanoLuc expression, and found that the P_RL3_ promoter induced the highest level of reporter expression (Figure 2D). To improve the sensitivity of photosensitive proteins, we engineered chimeric photosensory proteins BphP (PnBphP or FnBphP) by fusing the NTE (N‐terminal extension) of phytochrome A (PhyA) from *Arabidopsis thaliana* and the fungal phytochrome FphA from *Aspergillus nidulans* to the N‐terminus of *Dr*BphP‐PCM because PhyA's NTE was reported to stabilize the active far‐red light‐absorbing state.^[^ ^43^, ^44^, ^45^ ^]^ Our findings demonstrate that the RID system based on FnBphP and its interaction partner LDB3 supports highly efficient NanoLuc reporter expression upon RL illumination (Figure 2E).

### 2.2 Characterization of Immune Adjuvant Release by RIVA

Assessment of RID system performance showed that NanoLuc reporter gene expression was RL illumination time‐ and intensity‐dependent (Figure S2A,B, Supporting Information). To test its ON/OFF function, iPSCs transfected with this RID system were exposed to RL (660 nm, 2.0 mW cm^−2^) for 10 s to induce NanoLuc expression, followed immediately by exposure to FRL (780 nm, 1.0 mW cm^‐^^2^) for 2 min: an obvious NanoLuc signal was only observed in cells exposed to RL illumination; no signal was evident for cells first exposed to RL and then to FRL (Figure S2C, Supporting Information). Thus, the RID system can be switched ON with RL and switched OFF with FRL.

There was no obvious background increase over time under dark conditions; nor was there any increase in the NanoLuc signal after 24 h of stopping RL illumination (Figure S2D, Supporting Information). Further, this RID system exhibited obvious NanoLuc signal under RL illumination, while no induced gene expression was observed under different light wavelengths, highlighting the chromatic specificity (660 nm wavelength) of this RID system (Figure S2E, Supporting Information). In addition, this RID system enabled reversible control of the reporter gene expression (Figure S2F, Supporting Information).

**Figure 3:**
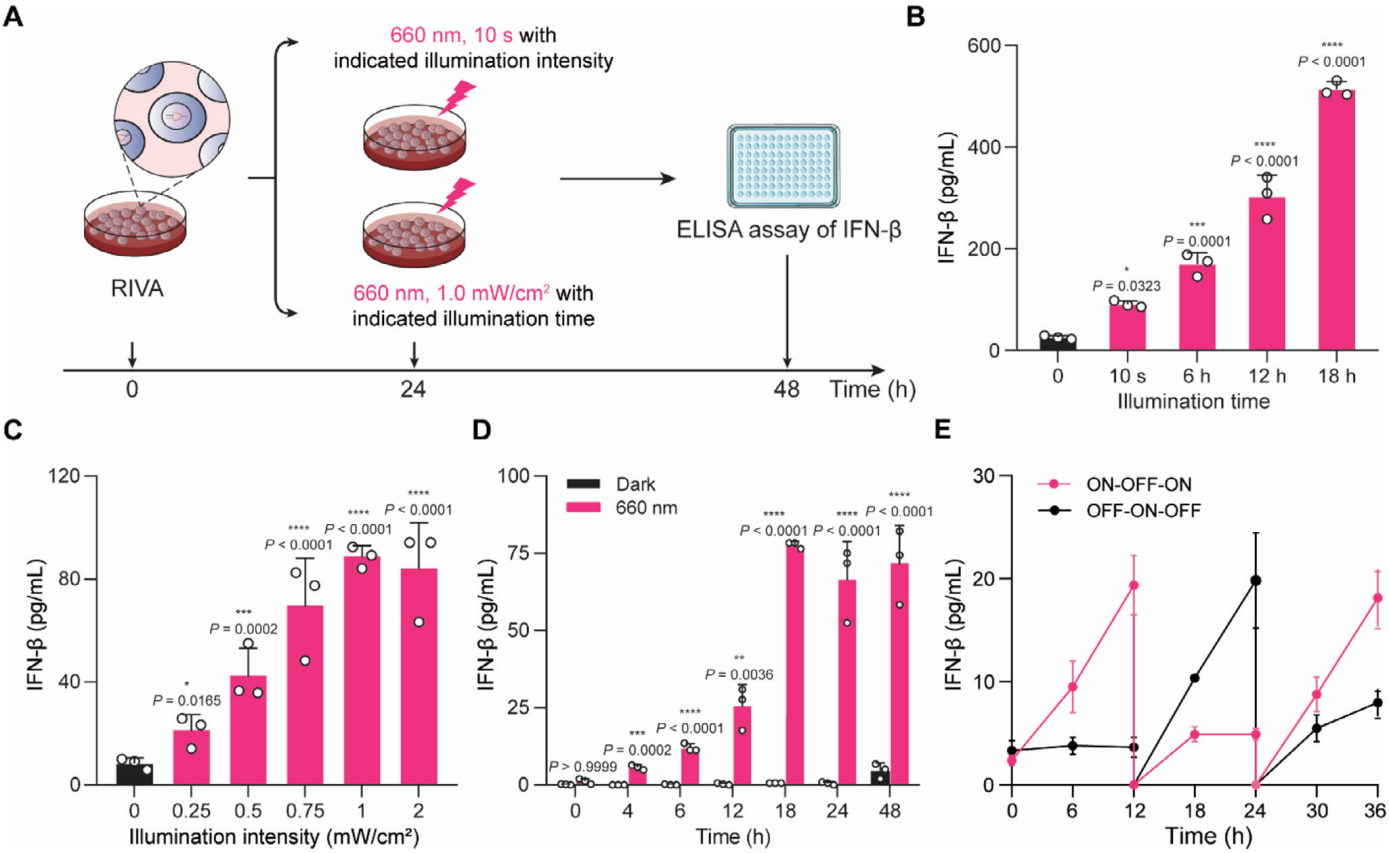
Characterization of IFN‐β release mediated by RIVA. A) Schematic of the experimental procedure for exposure time‐dependent and exposure intensity‐dependent IFN‐β release of RIVA. B) Exposure time‐dependent immune adjuvant (IFN‐β) release of RIVA. RIVA (2 × 10^4^) was illuminated with RL (1.0 mW cm^‐^^2^, 660 nm) for the indicated time points (0 to 18 h); culture supernatants were collected to quantify IFN‐β production using a mouse IFN‐β enzyme‐linked immunosorbent assay (ELISA) kit at 24 h after the initial illumination. C) Illumination‐intensity‐dependent IFN‐β release of RIVA. RIVA (2 × 10^4^) was illuminated with RL at the indicated light intensities (0 – 2.0 mW cm^‐^^2^) for 10 s, and culture supernatant was collected to quantify IFN‐β production as described in B. D) Quantification of RIVA mediated IFN‐β release kinetics. RIVA (2 × 10^4^) was illuminated with RL (660 nm, 1.0 mW cm^‐^^2^) for 10 s. IFN‐β production in the culture supernatant was quantified at the indicated time points (0 – 48 h) after illumination. E) Reversibility of immune adjuvant IFN‐β release by RIVA. RIVA cells (2 × 10^4^) was seeded into a 24‐well plate. After 24 h, the cells were either maintained in darkness (OFF) or illuminated with red light (RL, 0.5 mW cm^‐^^2^) for 10 s (ON). The experimental protocol involved alternating 12‐hour cycles of ON and OFF states, with IFN‐β production quantified every 6 h over a total period of 36 h. The culture medium was replaced every 12 h. Data in B–E are presented as means ± SD; *n* = 3 independent experiments. *P* values in B and C were calculated by one‐way ANOVA with a Turkey's test. *P* values in D were calculated by two‐tailed unpaired Student's *t*‐test. ns, not significant, *p < 0.05, **p < 0.01, ***p < 0.001, ****p < 0.0001.

Subsequently, we generated RIVA by stably transfecting iPSCs with plasmids carrying the light‐dependent transactivator LDB3‐p65‐HSF1 and a single vector which concatenated the constructs for RL‐responsive sensor Gal4 DBD‐FnBphP and the P_RL_‐driven immune adjuvant IFN‐β expression module. (Figure S3A, Supporting Information). Next, we first screened various RIVA cells by NanoLuc expression and evaluated the production profile of released IFN‐β, we found that clone no. 36 displayed high immune adjuvant release profile with more than 20‐fold induction upon RL illumination (Figure S3B,C, Supporting Information). We found that RIVA produces IFN‐β in a RL illumination intensity‐ and exposure‐time dependent manner (**Figure** **3**A–C), doing so without obvious increase in background signal over time under dark conditions (Figure 3D). Moreover, IFN‐β production from RIVA was reversible (Figure 3E). Thus, this result indicates that RIVA allows for robust, wavelength‐specific, adjustable, and reversible immune adjuvant expression.

### 2.3 Antitumor Effects of Therapeutic RIVA in Multiple Murine Tumor Models

Having confirmed the controllable release of IFN‐β from RIVA in vitro, we next evaluated its release capability in vivo using mitomycin C to suppress iPSC‐derived teratomas (Figure S4, Supporting Information). Wild‐type and A20 lymphoma‐bearing mice were randomly divided into four experimental groups: 1) phosphate‐buffered saline (PBS), 2) iPSCs alone, 3) RIVA without RL illumination (RIVA‐Dark), and 4) RIVA with RL illumination at 660 nm (RIVA‐660 nm). One day post‐vaccination, mice in the RIVA‐660 nm group were exposed daily to RL at an intensity of 20 mW cm^‐^^2^ for 10 min over a period of four consecutive days. Peripheral blood samples were subsequently collected for IFN‐β quantification. The results demonstrated that the RIVA‐660 nm group exhibited significantly elevated IFN‐β levels compared to the three control groups, confirming efficient and sustained IFN‐β release triggered by RL illumination for at least four days (Figure S5, Supporting Information).

**Figure 4:**
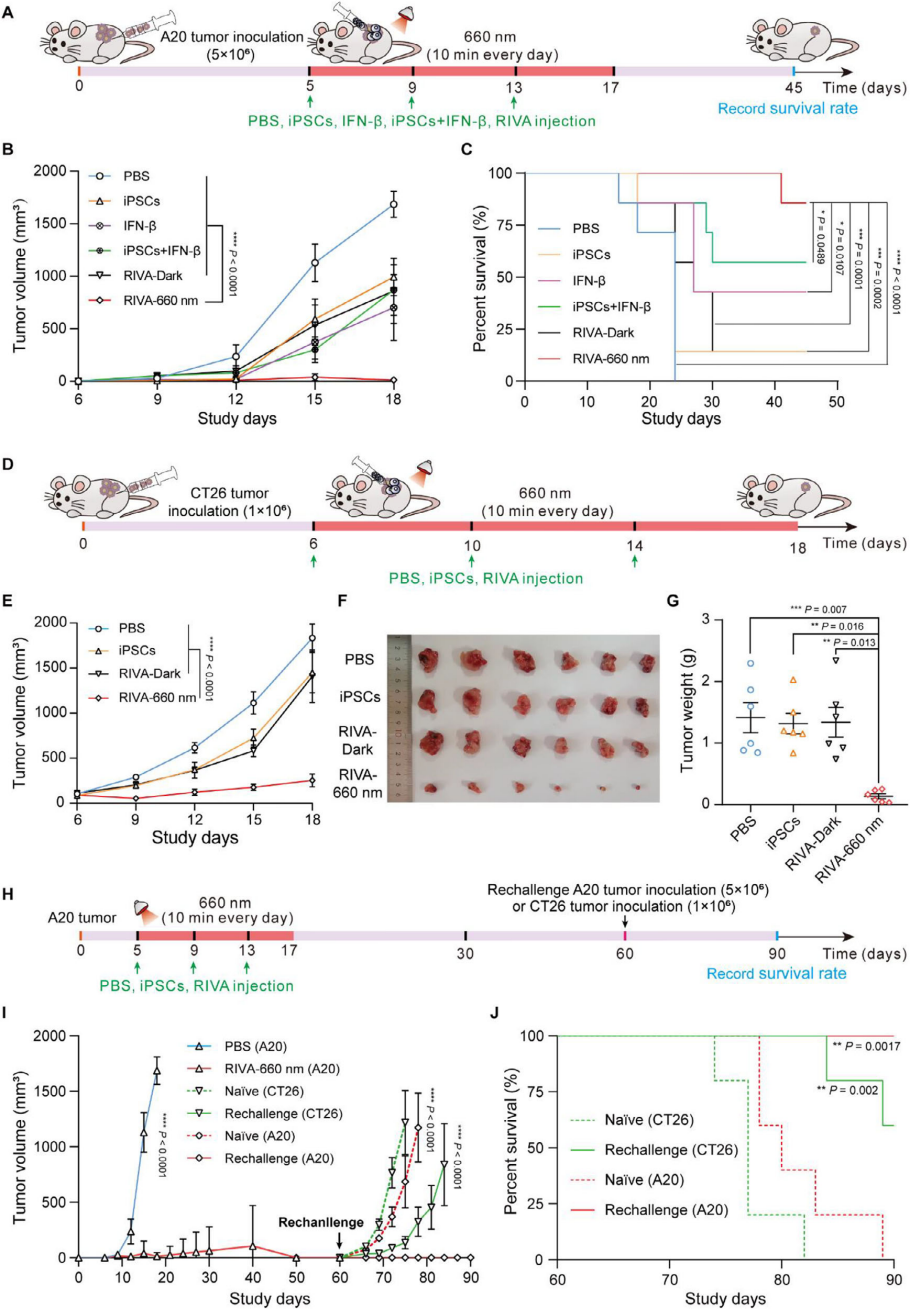
Evaluation of the antitumor effects of the therapeutic RIVA in multiple murine tumor models. A) Experimental procedure for evaluating the therapeutic performance of RIVA in a A20 lymphoma murine model. The A20 lymphoma murine model was established by subcutaneous injection of 5 × 10^6^ A20 cells into the dorsum of female BALB/c mice. After the tumor volume reached 50–80 mm^3^, the mice were intratumorally injected with RIVA (3 × 10^6^) three times at 4‐day intervals, and mice were then illuminated with or without RL (20 mW cm^‐^^2^) for 10 min each day for 12 days (RIVA‐660 nm group or RIVA‐Dark group). Mice injected with wild‐type iPSCs (3 × 10^6^), or IFN‐β (200 ng per mouse), or iPSCs + IFN‐β or PBS were examined as controls. All injections were administered once every 4 days for a total of 3 injections over the 12‐day period. B) The tumor volume measurement every 3 days in different mouse groups. C) Kaplan‐Meier survival curves of mice in different treatment groups in the A20 lymphoma murine model (B, C, *n* = 7–12 mice per group). D) Experimental procedure for evaluating the therapeutic performance of RIVA in a CT26 tumor model (as in A). E) Tumor volumes were measured every 3 days in the indicated different mouse groups. F) Images of dissected tumors and G) dissected tumor weights for the indicated mouse groups (D‐G, *n* = 6 mice per group). H) Experimental procedure for tumor rechallenge with A20 or CT26 cells. On day 60, the RIVA‐vaccinated mice (i.e., the RIVA‐660 nm group) that had remained tumor free were re‐challenged by subcutaneous injection of A20 cells (2.5 × 10^6^) or CT26 colon cancer cells (1 × 10^6^) on the contralateral flank, as were naïve, age‐matched controls. I) Tumor volume measurement in the indicated mouse groups. J. Kaplan‐Meier survival curves of mice in the indicated treatment groups (I, J, *n* = 5–12 mice per group). Data are presented as means ± SEM. *P* values in B, E, and I were calculated by two‐way ANOVA with a Sidak's test. *P* values in C and J were calculated by log‐rank (Mantel‐Cox) test. *P* values in G were calculated by one‐way ANOVA with a Turkey's test.

We next assessed the antitumor effect of RIVA in a murine A20 lymphoma model established by subcutaneous injection of A20 cells on day 0. Female BALB/c mice were divided into six experimental groups: 1) PBS, 2) iPSCs only, 3) IFN‐β only, 4) iPSCs + IFN‐β, 5) RIVA without RL illumination (RIVA‐Dark), and 6) RIVA with RL illumination (RIVA‐660 nm). Vaccination commenced on day 5 and was repeated twice at 4‐day intervals with the respective treatments (**Figure** **4**A).

RL illumination of the RIVA‐660 nm group for 10 min each day for 12 days resulted in a stronger tumor inhibitory effect against tumor growth and the longer survival rate compared to the PBS, RIVA‐Dark, iPSCs + IFN‐β, free IFN‐β, and iPSCs groups (Figure 4B,C). Notably, RIVA‐660 nm exhibited superior tumor suppression relative to iPSCs combined with an equivalent dose of free recombinant IFN‐β (Figure 4B). This enhanced efficacy is attributed to the limited half‐life of free IFN‐β, restricting its retention beyond 4 days post‐injection, whereas the RIVA‐660 nm group provided sustained IFN‐β release over the 4‐day interval, thus ensuring continuous antigenic interaction. Collectively, these results confirm that the RIVA system, by integrating multiple iPSC‐derived antigens with immune adjuvants under RL illumination, confers potent protection against tumor recurrence.

To further examine the immune responses associated with the antitumor effects of RIVA, we utilized an A20 lymphoma‐bearing mouse model. Mice were again divided into six treatment groups: 1) PBS, 2) iPSCs only, 3) IFN‐β only, 4) iPSCs + IFN‐β, 5) RIVA‐Dark, and 6) RIVA‐660 nm. Peripheral blood and tumor tissues were collected on day 22 post‐tumor inoculation to assess immune activity (Figure S6A, Supporting Information). The proportions of mature dendritic cells (DCs, CD86^+^/MHC class II^+^), CD4^+^CD3⁺ T cells, and CD8^+^CD3⁺ T cells were significantly higher in both peripheral blood and tumor tissues from the RIVA‐660 nm group compared to control groups (Figure S6B–D and F–H, Supporting Information). Additionally, there was a significant increase in natural killer (NK) cells (CD49b^+^) in the RIVA‐660 nm group (Figure S6E,I, Supporting Information). These findings collectively highlight that the antitumor effects mediated by RIVA are driven by robust immune activation involving mature DCs, CD4^+^ and CD8^+^ T cells, and NK cells.

The CT26 model is weakly immunogenic. It responds poorly to immunotherapy.^[^ ^46^, ^47^ ^]^ To further demonstrate the therapeutic potential of our RIVA, we tested the tumor inhibitory effect in the murine CT26 tumor model (Figure 4D). Similar to that observed for A20 tumors, tumor growth assessment by caliper measurement and tumor weights showed that significant inhibition tumor growth was observed for the RIVA‐660 nm group; no such inhibition was observed for dark control RIVA immunized mice and to other control mice (Figure 4E–G). Therefore, these results establish that the therapeutic RIVA (under RL illumination) confer strong tumor inhibitory effects in different types of tumors.

### 2.4 RIVA Protects Against Tumor Rechallenge in the A20 Lymphoma Murine Model

To investigate whether the RIVA‐660 nm group could provide long‐term protection beyond 60 days in a A20 lymphoma tumor re‐challenge model, the cured mice of RIVA‐660 nm group were inoculated with the original A20 cells or CT26 colon cancer cells at day 60 (Figure 4H). The immunized mice inoculated with original A20 cells showed no detectable tumor masses and a significant prolonged mouse survival rate of 100% within 90 days. In contrast, the naïve mice inoculated with A20 cells showed obvious tumor growth after re‐challenge. Similar to that observed for A20 tumors, the immunized mice inoculated with another CT26 cells showed apparent anti‐tumor effect compared with the naïve mice inoculated with CT26 cells (Figure 4I,J). Together, these results suggest that RIVA (under RL illumination) is capable of achieving long‐lived anti‐tumor responses.

To evaluate the biosafety of RIVA, at the end of the tumor rechallenge experiment in the A20 lymphoma tumor mode, whole blood was collected and subjected to routine blood test [including white blood cells (WBC), red blood cells (RBC), hemoglobin (HGB), hematocrit (HCT), mean corpuscular volume (MCV), mean corpuscular hemoglobin (MCH), mean corpuscular hemoglobin concentration (MCHC), platelets (PLT), platelet crit (PCT)] (Figure S7A–I, Supporting Information) and serum biochemistry analyses [including liver enzymes alanine aminotransferase (ALT), aspartate aminotransferase (AST), surrogate of kidney function blood urea nitrogen (BUN), and creatinine (CRE)] (Figure S7J–M, Supporting Information). There were no obvious differences observed in any of the tested blood examination indicator between wild type control mice and RIVA‐660 nm group (Figure S7A–M, Supporting Information). Moreover, hematoxylin and eosin (H&E) staining revealed that no obvious histological changes were observed in any of the examined organs including heart, liver, spleen, lung, and kidney (Figure S8, Supporting Information). Of note, we also found that the serum levels of tumor necrosis factor‐α (TNF‐α) and interlukine‐6 (IL‐6), two representative cytokines for cellular immunity, were the highest in mice given the iPSCs constitutively expressing IFN‐β due to the overstimulated immune response among all the groups (Figure S9, Supporting Information). These results suggest that the RIVA have a favorable biosafety profile without inducing a cytokine storm.

### 2.5 Prophylactic Effect of RIVA in Multiple Murine Tumor Models

**Figure 5:**
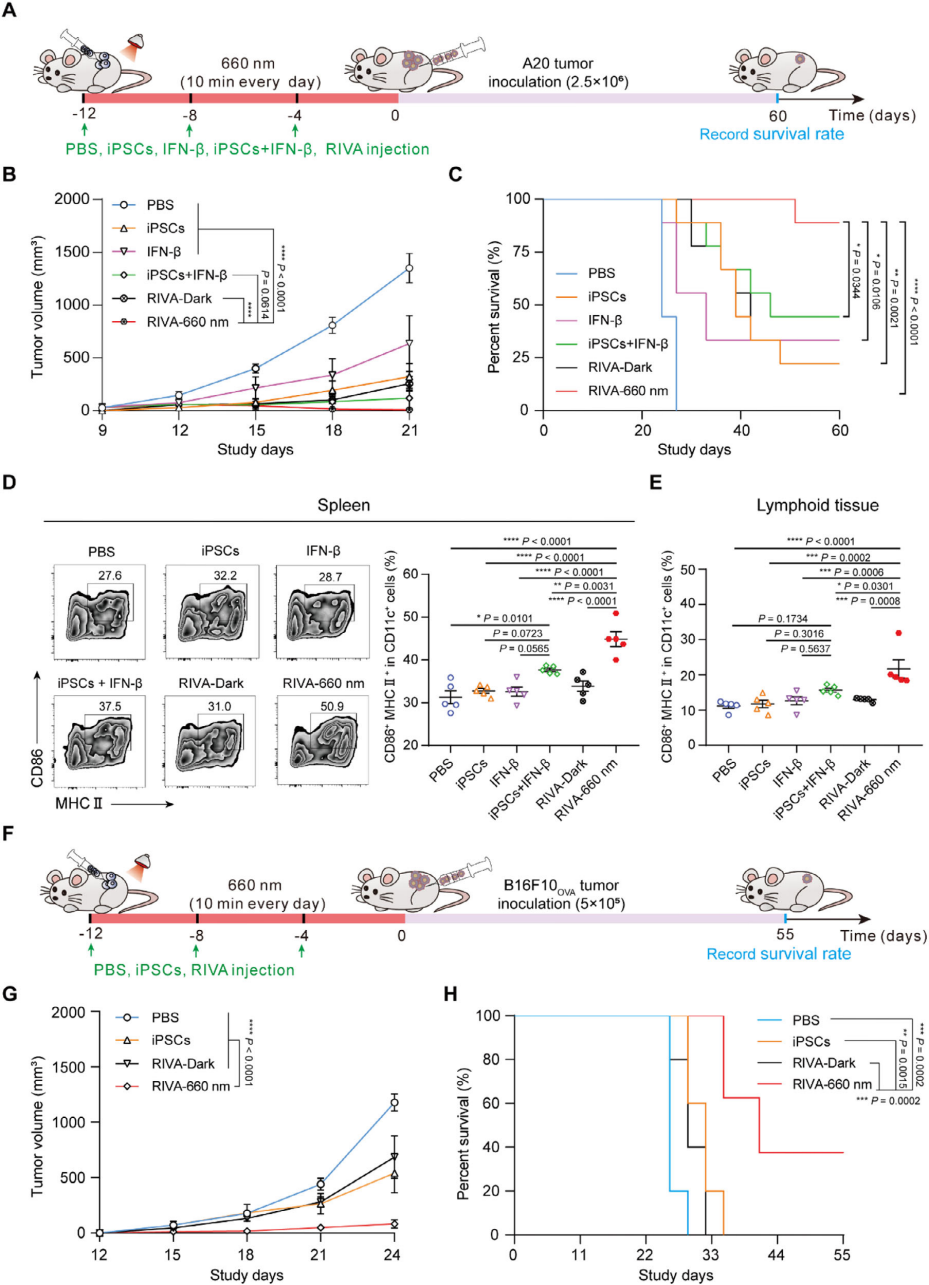
Evaluation of the prophylactic effect of RIVA in two murine tumor models. A) Experimental procedure for evaluating the performance of RIVA in a prophylactic A20 lymphoma murine model. The female BALB/c mice were subcutaneously inoculated with RIVA (3 × 10^6^) three times at 4‐day intervals; mice were then illuminated with or without RL (20 mW cm^‐^^2^) for 10 min each day for 12 days (RIVA‐660 nm group or RIVA‐Dark group). Mice injected with wild‐type iPSCs (3 × 10^6^), or IFN‐β (200 ng per mouse), or iPSCs + IFN‐β or PBS were examined as controls. After 12 days, the immunized mice were subcutaneously injected with 5 × 10^6^ A20 cells. B) Tumor volume measurement every 3 days in the indicated mice groups. C) Kaplan‐Meier survival curves of mice in different treatment groups in the prophylactic A20 lymphoma murine model (B, C, *n* = 9 mice per group). D, E. Assessing the matured DC cells in the spleens D) and lymphatic nodes E) of mice sacrificed on day 7 after tumor inoculation by flow cytometry. Representative flow cytometry zebra plot (left) and corresponding quantification (right) of CD86^+^MHCII^+^ DCs. (D, E, *n* = 5 mice per group). F) Schematic showing the experimental procedure for evaluating RIVA performance in a prophylactic B16F10_OVA_ tumor model (as in A). G) Tumor volume measurement every 3 days in the indicated mouse groups. H) Kaplan‐Meier survival curves of mice in different treatment groups in the B16F10_OVA_ tumor model (G, H, *n* = 5–8 mice per group). Data are presented as means ± SEM. *P* values in B and G were calculated by two‐way ANOVA with a Sidak's test. *P* values in C and H were calculated by log‐rank (Mantel‐Cox) test. *P* values in D and E were calculated by one‐way ANOVA with a Turkey's test.

To investigate the effectiveness of RIVA in a prophylactic model, female BALB/c mice were first injected subcutaneously with 1) PBS, 2) iPSCs only, 3) IFN‐β only, 4) iPSCs + IFN‐β, 5) RIVA‐Dark, and 6) RIVA‐660 nm once each four days (over 12 days), followed by subcutaneous injection of 2.5 × 10^6^ A20 cells on day 0. Tumor size was measured by calipers every 3 days, and mouse survival was assessed at the pre‐determined end‐point of 60 days (**Figure** **5**A). RIVA with RL illumination for 10 min each day significantly inhibited tumor growth compared to the dark control RIVA immunized mice and to the control groups (Figure 5B); the illuminated RIVA group also had prolonged survival (90% survival after 60 days) (Figure 5C).

DCs are primary antigen‐presenting cells (APCs) that orchestrate both innate and adaptive immune responses.^[^ ^48^, ^49^, ^50^ ^]^ We next examined the ability of RIVA to induce DC maturation. Flow cytometric analysis of spleen and lymph node samples showed that the proportion of mature DCs within the total DC population (CD11c^+^) was significantly increased in the RIVA‐660 nm group compared to control groups (Figure 5D,E). These findings support that RIVA effectively promotes DC maturation in the spleen and lymph nodes under RL illumination.

We also performed similar studies on C57BL/6 mice inoculated with B16F10 ovalbumin (B16F10_OVA_) expressing melanoma cells (Figure 5F). The RIVA immunized mice that received RL illumination exhibited significantly inhibited tumor growth compared to control mice (Figure 5G) and had prolonged survival (Figure 5H). Collectively, these results support that RIVA confers prophylactic benefit against distinct murine tumor models (A20 and B16F10_OVA_) in mice.

### 2.6 Anti‐Tumor Immune Response Elicitation by RIVA

**Figure 6:**
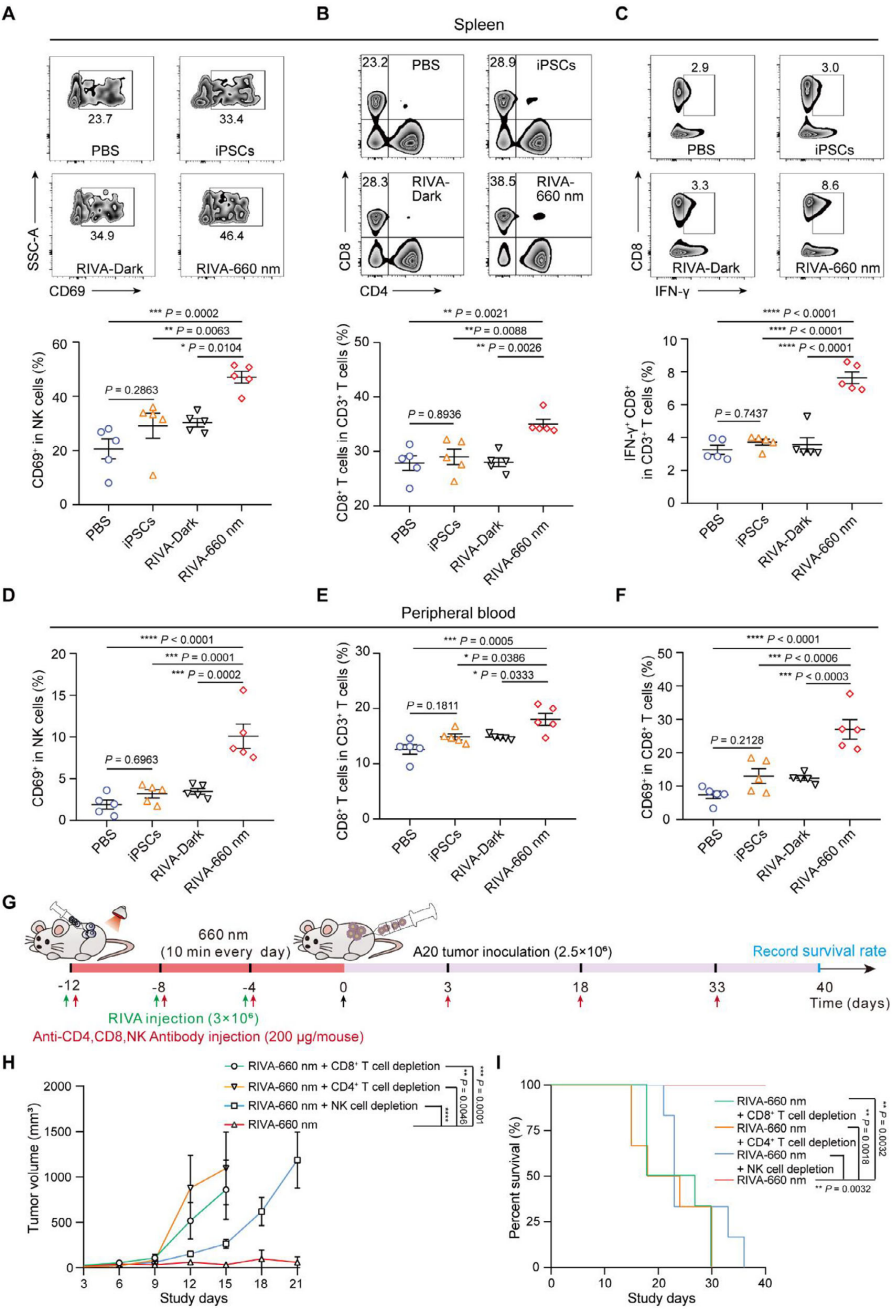
Anti‐tumor immune responses elicited by RIVA in a prophylactic model. Female BALB/c mice were subcutaneously injected with RIVA (3 × 10^6^) three times at 4‐day intervals and illuminated with or without RL (20 mW cm^‐^^2^) for 10 min each day for 12 days (RIVA‐660 nm group or RIVA‐Dark group). Mice injected with wild‐type iPSCs (3 × 10^6^) or PBS were examined as controls. After 12 days, the immunized mice were subcutaneously injected with 5 × 10^6^ A20 cells. Spleens were removed and ground to collect lymphocytes on day 7 after injection of A20 cells for flow cytometry analysis. The activated phenotypes of NK cells and CD8^+^ T cells evaluated in spleen of mice sacrificed on day 7 by flow cytometry. Representative flow cytometric zebra plot (above) and corresponding quantification (below) of CD69^+^ NK cells A) and CD8^+^ T cells B). C) The percentage of IFN‐γ^+^CD8^+^ T cells among splenic lymphocytes. Splenocytes were cultured with cell stimulation cocktails containing phorbol 12‐myristate 13‐acetate, ionomycin, brefeldin A and monesin at 37 °C. After 6 h, IFN‐γ^+^CD8^+^ T cells were analyzed by flow cytometry. Representative flow cytometry zebra plot (above) and corresponding quantification (below) of IFN‐γ^+^CD8^+^ T cells. D–F) The activated NK cells and CD8^+^ T cells evaluated in peripheral blood of mice sacrificed on day 7 by flow cytometry. Quantification of CD69^+^ NK cells (D), CD8^+^ T cells (E), and CD69^+^CD8^+^ T cells (F) in peripheral blood. G) Schematic showing the experimental procedure for investigating anti‐tumor immunity in the prophylactic A20 lymphoma murine model. Female BALB/c mice were subcutaneously injected with RIVA (3 × 10^6^) and intraperitoneally injected with NK, CD8α, or CD4 depleting antibodies (200 µg/mouse) three times at 4‐day intervals; mice were then illuminated with RL (20 mW cm^‐^^2^) for 10 min each day for 12 days (RIVA‐660 nm group). After 12 days, the immunized mice were subcutaneously injected with 5 × 10^6^ A20 cells and intraperitoneally injected with NK, CD8α, or CD4 depleting antibodies (200 µg/mouse) every two weeks (three doses in total). H) Tumor growth in the control (NK, CD8α, or CD4 depleting antibody) and RIVA‐660 nm group. I) Kaplan‐Meier survival curves of mice. Data in A‐F are presented as the means ± SEM (*n* = 5 mice per group). Data in H and I are presented as the means ± SEM (*n* = 6 mice per group). *P* values in A–F were calculated by one‐way ANOVA with a Turkey's test. *P* values in H were calculated by two‐way ANOVA with a Sidak's test. *P* values in I were calculated by log‐rank (Mantel‐Cox) test.

NK function in anti‐tumor immunity by recognizing and killing tumor cells without prior sensitization or activation, while CD4^+^ T cells and CD8^+^ T cells mediate adaptive immune responses. We profiled the immune responses ostensibly underlying the observed anti‐tumor effects of RIVA by using flow cytometry to monitor the immune cell components of spleen and peripheral blood samples from the aforementioned prophylactic A20 model for 4 sample groups (sampled on day 7): 1) PBS, 2) iPSCs only, 3) RIVA‐Dark, and 4) RIVA‐660 nm. The percentage of NK cells (CD69^+^CD49b^+^) was significantly increased in the RIVA‐660 nm group compared with the various control groups (**Figure** **6**A,D; Figure S10, Supporting Information), and the RIVA‐660 nm group had significantly higher percentages of CD8^+^CD3^+^ T cells (Figure 6B,E) and of CD4^+^CD45^+^ T cells (Figure S11, Supporting Information). In addition, we assessed CD8^+^ T cell functionality by measuring IFN‐γ production after *ex vivo* re‐stimulation (with Cell Stimulation Cocktail). We observed that the RIVA‐660 nm group had significantly increased percentages of IFN‐γ^+^CD8^+^ T cells and CD69^+^CD8^+^ T cells (among total CD8^+^ T cells) as compared with the control groups (Figure 6C,F).

We also performed T cell and NK cell depletion experiments using neutralizing antibodies against cell surface markers in the prophylactic A20 cell model mice. Whereas obvious tumor growth was evident for the RIVA immunized mice (with RL illumination) given antibodies to block NK cells or CD4/CD8 T cells (Figure 6G), no tumors were observed in mice lacking antibody‐mediated immune cell depletion (Figure 6H). Moreover, the overall survival rates of mice in the three depletion groups decreased remarkably (Figure 6I). These results establish that RIVA elicits innate immune activation effects through NK cells and elicits adaptive immune anti‐tumor responses through CD4^+^ and CD8^+^ T cells.

### 2.7 Anti‐Metastatic Effect of RIVA in a Murine 4T1 Tumor Model

**Figure 7:**
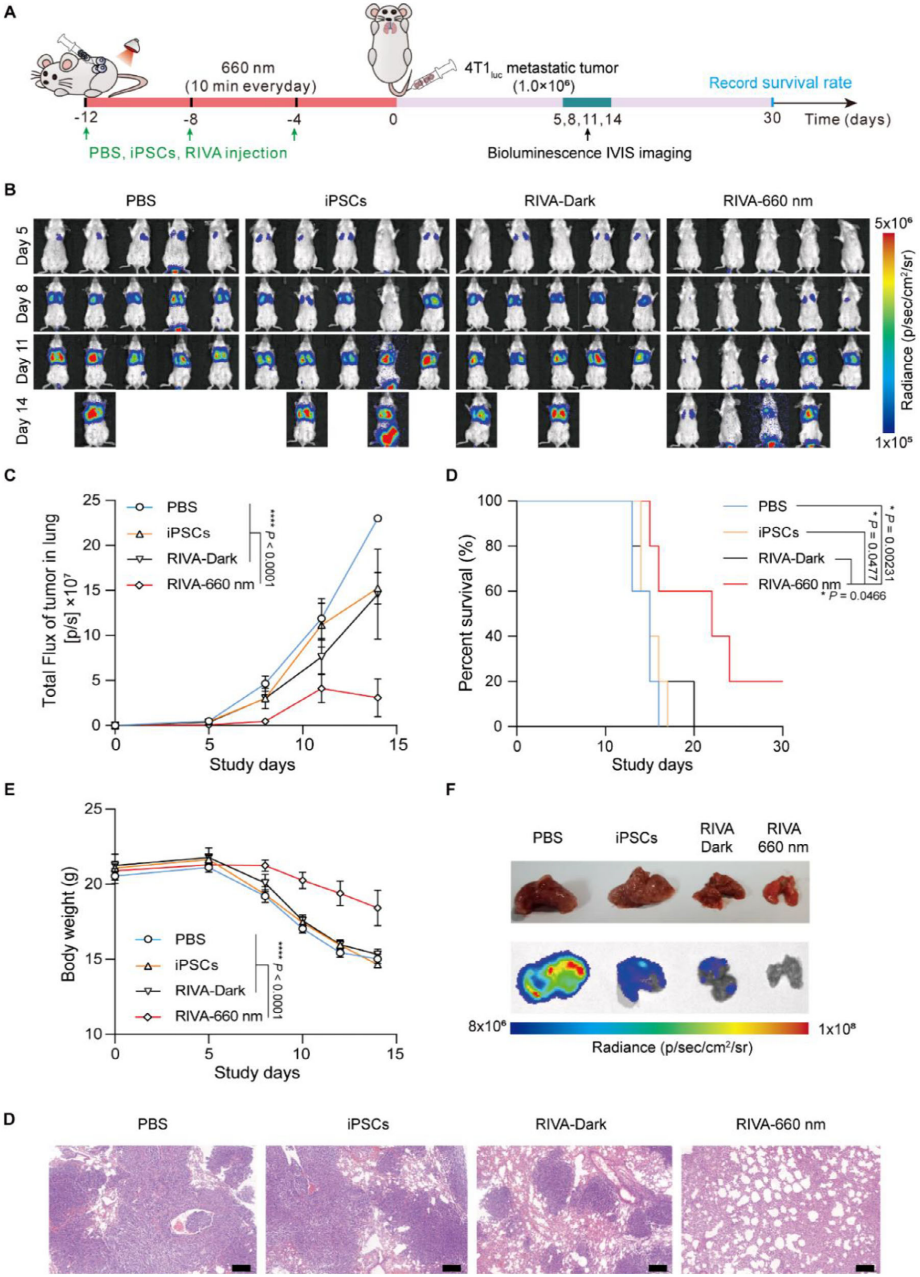
Anti‐metastatic effect of RIVA in a murine 4T1 tumor model. A) Experimental procedure for evaluating RIVA performance in a 4T1_luc_ pulmonary metastasis model. Female BALB/c mice were subcutaneously injected with RIVA (3 × 10^6^) three times at 4‐day intervals; mice were then illuminated with or without RL (20 mW cm^‐^^2^) for 10 min each day for 12 days (RIVA‐660 nm group or RIVA‐Dark group). Mice injected with wild‐type iPSCs (3 × 10^6^), or PBS were examined as controls. After 12 days, the immunized mice were intravenously injected with 1 × 10^6^ murine 4T1_luc_ breast tumor cells. B) In vivo bioluminescence imaging of 4T1_luc_ lung metastasis in different mice groups. C) Quantification of tumor burden based on the bioluminescence IVIS imaging from B. D) Kaplan‐Meier survival curves and E) body weights of mice in the indicated mouse groups. F) Dissected lungs of the indicated mouse groups. G) Images of H&E‐stained lung slides. Scale bars, 200 µm. Data in C‐E are presented as means ± SEM (*n* = 5 mice per group). *P* values in C and E were calculated by two‐way ANOVA with a Turkey's test. *P* values in D were calculated by log‐rank (Mantel‐Cox) test.

We also investigated RIVA‐enabled modulation of antitumor immune responses in a model based on murine 4T1 tumor cells, a highly tumorigenic and invasive cancer.^[^ ^51^, ^52^ ^]^ Female BALB/c mice were first subcutaneously injected with 1) PBS, 2) iPSCs only, 3) RIVA‐Dark, or 4) RIVA‐660 nm once each four days (over 12 days), followed by intravenously injected with 1 × 10^6^ murine 4T1_luc_ breast tumor cells on day 0 (**Figure** **7**A). Bioluminescent IVIS imaging evaluating the luciferase signals (based on the bioluminescence signals of 4T1_luc_ cells) showed that the RIVA‐660 nm group had significantly fewer luciferase signals in the lungs compared to control animals (PBS group, or iPSCs group, or RIVA‐Dark group) on days 5, 8, 11, and 14 (Figure 7B,C). Moreover, the RIVA‐660 nm group had markedly extended animal survival over the other groups (Figure 7D). No obvious decrease in the mice body weight was detected during vaccine treatment in the RIVA‐660 nm group (Figure 7E).

We also harvested and photographed lungs on day 11. In the RIVA‐660 nm group, the lung metastases were reduced. In contrast, dense metastases were obvious in the control animals (Figure 7F). H&E staining also supported that the RIVA‐660 nm group had a reduced number of the metastatic nodules in the lungs (Figure 7G). Together, these results indicate that RIVA had preventive effects in the highly tumorigenic and invasive cancers

## 3 Discussion

Seeking to improve the immune effect of an iPSC‐based cancer vaccines while minimizing the risk of systemic toxicity, we here developed the RIVA using the chimeric photosensory protein FnBphP and its interaction partner LDB3. RIVA harnesses the multiple antigens from iPSCs while enabling optogenetic control of IFN‐β expression under RL illumination. RIVA inhibited tumor growth and prolonged mouse survival in multiple tumor models, including A20 lymphoma, CT26 colorectal carcinoma, B16F10 melanoma, and a metastatic 4T1 model. We experimentally confirmed that RIVA stimulates both innate and adaptive immune responses in mice, doing so without inducing a cytokine storm.

Cytokines are viewed as natural adjuvant candidates for increasing or modulating anti‐tumor effects.^[^ ^11^, ^53^, ^54^, ^55^ ^]^ However, their systemic administration often leads to uncontrolled distribution and severe inflammatory side effects.^[^ ^26^, ^58^, ^59^, ^60^ ^]^ To address this, we engineered iPSCs using the RID system to enable red light–inducible expression of IFN‐β. This optogenetic control significantly reduced systemic inflammation, as evidenced by lower serum levels of TNF‐α and IL‐6 compared to iPSCs constitutively expressing IFN‐β (Figure S9, Supporting Information), thereby avoiding overstimulation of the immune response.

Although both the iPSCs + IFN‐β group and the RIVA‐660 nm group (Figure 4B and Figure 5B) exhibited antitumor effects, the therapeutic efficacy of RIVA was markedly superior. This disparity primarily stems from differences in pharmacokinetics and delivery strategy. Recombinant IFN‐β administered systemically has a short serum half‐life and is rapidly cleared from circulation, limiting its therapeutic window and diminishing synergy with iPSC‐derived antigens. In contrast, RIVA enables spatiotemporal control of IFN‐β expression, sustaining localized cytokine availability at the tumor site to drive a more effective and durable immune response. Beyond cancer immunotherapy, the modularity of this optogenetic platform allows for broader translational applications. The RID system could be adapted to control the expression of other therapeutic proteins, such as immune adjuvants for infectious diseases or biologics like enzymes, peptide hormones, and antibodies. For instance, it may be used to regulate urate oxidase expression for gout treatment,^[^ ^61^ ^]^ or parathyroid hormone (PTH)^[^ ^62^ ^]^ for managing hyperparathyroidism, offering a flexible and responsive approach to precision medicine.

Despite the demonstrated efficacy of engineered optogenetic iPSC‐based cancer vaccines in preventing tumor progression across multiple murine cancer models, it is noteworthy that RIVA exhibited slightly reduced effectiveness against weakly immunogenic tumor models (*e.g*., CT26 colorectal carcinoma and B16F10 melanoma) compared to strongly immunogenic models (*e.g*., A20 lymphoma). This observation may result from the immunosuppressive nature of the tumor microenvironment, which typically comprises tumor cells, vascular endothelial cells, and various tumor‐infiltrating immune cell populations.^[^ ^63^, ^64^, ^65^ ^]^ Future investigations combining RIVA with checkpoint inhibitors such as anti‐PD‐1 or anti‐CTLA‐4 antibodies, or chemokines, might significantly enhance antitumor efficacy in these weakly immunogenic tumor contexts.^[^ ^66^, ^67^ ^]^ Moreover, given the greater complexity inherent to human malignancies relative to murine models, further validation of RIVA through additional preclinical studies and ultimately clinical trials will be essential to fully evaluate its therapeutic potential.

In the tumor models, IFN‐β release from RIVA‐iPSCs gradually decreased over four days without the 780 nm light illumination. As such, an explicit “OFF” signal was not required in these specific in vivo settings. However, an ON–OFF switch remains a critical design feature for broader therapeutic applications. This functionality enables temporal control over therapeutic protein production, allowing clinicians to modulate treatment according to disease progression, adverse events, or patient‐specific needs. For example, in the case of metabolic diseases like diabetes, precise control over insulin release is essential. In such contexts, the ability to both activate and deactivate the system on demand would be indispensable for maintaining physiological homeostasis and preventing hypoglycemia.

Our study establishes a proof‐of‐principle for the development of the optogenetic iPSC‐based cancer vaccines platform with the ability to activate the desired immune response. We envision that this strategy provides a controllable platform for effectively manipulating the immune response for any immunotherapeutic agents.

## 4 Conclusion

In this study, we have developed an innovative cancer vaccine for cancer immunotherapy by developing RIVA. We ultimately show that RIVA preserves the multiple intrinsic tumor antigens of iPSCs while also enabling optogenetic control of the expression of the immune adjuvant IFN‐β under RL illumination. We demonstrate that RIVA inhibits tumor growth and increased animal survival by promoting activation of both innate and adaptive immune responses, with minimal systemic toxicity.

## 5 Experimental Section

### Ethical Statement

The experiments involving animals were performed according to the protocol approved by the East China Normal University (ECNU) Animal Care and Use Committee and were in direct accordance with the Ministry of Science and Technology of the People's Republic of China on Animal Care Guidelines. The protocol was approved by the ECNU Animal Care and Use Committee (protocol ID: m20220405). All mice were euthanized after the termination of the experiments.

### Cloning and Plasmid Construction

All plasmids used in this study are provided in Table S1 (Supporting Information) and the DNA sequences for RID components in this study were listed in Table S3 (Supporting Information). The plasmids were constructed by Gibson assembly according to the manufacturer's instruction (MultiS One Step Cloning Kit, Catalog no. C113‐01, Vazyme) and the sequences were confirmed by DNA sequencing (Shanghai Saiheng Biotechnology).

### Cell Culture and Transfection

Human embryonic kidney cells (HEK‐293T, ATCC: CRL‐11268) were cultured at 37 °C in a humidified atmosphere containing 5% CO_2_ in Dulbecco′s Modified Eagle Medium DMEM (Catalog no. 12 100 061, Gibco) containing 10% v/v fetal bovine serum (FBS, Catalog no. FBSSA500‐S, AusGeneX) and 1% v/v penicillin/streptomycin solution (Catalog no. ST488‐1/ST488‐2, Beyotime Biotechnology). Mouse embryonic fibroblast (MEF) was prepared from the E13.5 embryos of CD‐1 mouse and cultured in DMEM supplemented with 10% v/v FBS (Gibco; Catalog no. 16000–044), 1% (v/v) non‐essential amino acid (NEAA, Catalog no. 11140‐050, Gibco), and 1% v/v penicillin/streptomycin solution. Mouse iPSCs were maintained on irradiated mouse embryonic fibroblast feeder cells in Glasgow Minimum Essential Medium (GMEM, Catalog no. 11710‐035, Gibco) with 15% (v/v) FBS (Catalog no. 16000–044, Gibco), 1% (v/v) penicillin/streptomycin solution, 1% (v/v) GlutaMAX (Catalog no. 35050–061, Gibco), 1% (v/v) NEAA, 0.1 × 10^−3^ M β‐mercaptoethanol (Catalog no. M3148, Sigma–Aldrich), 1000 U mL^−1^ recombinant mouse leukemia inhibitory factor (LIF, Catalog no. ESG1107, Millipore), 3 µM CHIR99021(Catalog no. S2924, Seleckchem), and 1 µM PD0325901 (Catalog no. S1036, Seleckchem).

The melanoma cells (B16F10_OVA_, Catalog no. NM‐S31A‐TG05, Shanghai Model Organisms Center) and mammary carcinoma cells (4T1_luc_, Luciferase tagged by stable plasmid transfection, ATCC: CRL‐2539) were cultured in DMEM (Catalog no. 12 100 061, Gibco) containing 10% FBS (Catalog no. FBSSA500‐S, AusGeneX) and 1% (v/v) penicillin/streptomycin solution. The colon cancer cells (CT26, ATCC: CRL‐2638) were cultured in RPMI‐1640 (Catalog. no. 8 122 663, Gibco) containing 10% FBS (Catalog no. FBSSA500‐S, AusGeneX) and 1% (v/v) penicillin/streptomycin solution. The lymphoma cell (A20, ATCC: TIB‐208) were cultured in RPMI‐1640 containing 10% FBS (Catalog no. 2 440 076, Gibco), 1% (v/v) penicillin/streptomycin solution and 0.1 × 10^−3^ M β‐mercaptoethanol. All the cells were cultured at 37 °C in a humidified atmosphere containing 5% CO_2_ and were regularly tested for the absence of *Mycoplasma* and bacterial contamination. The concentration and viability of the cells were evaluated using a Countess II Automated cell counter (AMEP4746, Life Technologies).

Mouse iPSCs were transfected using Lipofectamine 3000‐based protocol. Briefly, 2 × 10^4^ cells per well were plated into a 24‐well plate and co‐transfected with corresponding plasmid mixtures using Lipofectamine 3000 (Catalog no. L3000015, Thermo Fisher Scientific) according to the manufacturer's instructions.

### Lentivirus Preparation

HEK‐293T cells (5 × 10^6^) were seeded into a 15 cm dish and cultured for 16 h before transfection and were subsequently co‐transfected with a plasmid encoding the desired gene of interests (15 µg), lentiviral package plasmid (psPAX2, 15 µg, Catalog no. 12 260, Addgene), and a plasmid encoding for VSV‐G pseudotyping coat protein (pMD2.G, 7.5 µg, Catalog no. 12 259, Addgene) using an optimized polyethyleneimine (PEI)‐based protocol with 1.5 mL of a 3:1 PEI:DNA mixture (w/w) (PEI, MW 40 000, stock solution 1 mg mL^−1^ in ddH_2_O; Catalog no. 24 765, Polysciences). After 6 h, media were changed. After 48 h post‐transfection, cell supernatants containing the virus were harvested and filtered through a 0.45 µm syringe filter (Catalog no. 4654, Pall Corporation). Then the filtered supernatants were concentrated by ultracentrifugation at 25 000 × *g* for 2 h at 4 °C using a Beckman Avanti JXN‐26 centrifuge with a JA‐20 rotor (Beckman Coulter, Inc.), and virus pellets were re‐suspended with 100 µL phosphate buffered saline (PBS) for immediate use or stored at −80 °C before use.

### Induction of Pluripotent Stem Cells

To generate iPSCs, MEFs were transduced with the lentiviral encoding Sox2 and Oct4. after 24 h transduction, the media were changed to MEF media. On the 3rd day, the media were changed to iPSCs media containing valproic acid (VPA, 0.5 mM, Sigma) once per 2 days. Clones became visible on day 7; on day 14–15, they were picked for further experiments.

### Characterization of iPSCs

The obtained iPSCs were identified by immunofluorescence staining. iPSCs were fixed with 4% w/v paraformaldehyde fix solution (PFA, Catalog no. E672002‐0500, Sangon Biotech) for 15 min, permeabilized with 0.2% Triton X‐100 (dissolved in PBS; Catalog no. A600198‐0500, Sigma) for 15 min, and then blocked with 10% v/v goat serum (Catalog no. C0265, Beyotime Biotechnology) for 30 min at room temperature. Subsequently, cells were incubated with primary antibodies overnight at 4 °C with goat polyclonal anti‐Sox2 (1:1000, Catalog no. AF2018, R&D systems), rabbit polyclonal anti‐Oct4 (1:400, Catalog no. 2840, Cell Signaling Technology), or mouse anti‐SEEA1 (1:100, Catalog no. 125 606, Biolegend). After washing with PBST (0.2% Triton X‐100 in PBS) for 5 min three times, cells were incubated with secondary antibodies of goat anti‐rabbit IgG (H+L) (Alexa Fluor 594; 1:200; Catalog no. 33112ES60, YEASEN Biotechnology), donkey anti‐goat IgG (H+L) (Alexa Fluor 594; 1:200; Catalog no. SDAG594, YiShan Biotech) for 1 h at room temperature. After washings with PBST for 5 min three times, the cell nuclei were counterstained with DAPI (5 µg mL^−1^, Catalog no. C1002, Beyotime Biotechnology) for 10 min and were observed by an inverted fluorescence microscope (DMI8, Leica).

iPSCs were further identified by flow cytometry. Cells were washed with PBS and incubated were stained with PE anti‐mouse SEEA1 (1:100, Catalog no. 125 606, Biolegend) or PE Mouse IgM, κ isotype control antibody (1:100, Clone MM‐30; Catalog no. 401 611, Biolegend) or for 30 min at room temperature. After washing with PBS for 5 min three times, cells were analyzed using a BD LSRFortessa Flow Cytometer (BD Biosciences). A minimum of 10 000 events per plot were collected and analyzed using the FlowJo V10.6.2 software.

### NanoLuciferase (NanoLuc) Reporter Assay

The NanoLuc activities were quantified using Nano‐Glo Luciferase Assay (catalog no. N1130, Promega) according to the manufacturer's instructions. Nano‐Glo Luciferase Assay reagent was prepared by combining one volume of Nano‐Glo Luciferase Assay substrate with 50 volumes of assay buffer. Then, 8.0 µL of cell culture supernatant was mixed with 8.0 µL substrate‐containing assay buffer in a white 384‐well plate (catalog no. 781 076, Greiner Bio One) and incubated for 5 min at room temperature. Luminescence intensity was measured by the Synergy H1 hybrid multi‐mode microplate reader (BioTek Instruments) with Gen5 software (version: 2.04).

### Generation of Stable Cell Lines

The stable cell line RIVA, transgenic for the constitutive RL‐inducible co‐expression of mouse IFN‐β, was constructed by co‐transfecting mouse iPSCs with pNX175 (ITR‐P_EF1α_‐2NLS‐LDB3‐p65‐HSF1‐pA::P_mPGK_‐PuroR‐pA‐ITR), pNX176 (ITR‐P_EF1α_‐Gal4 DBD‐FnBphP‐pA::P_RL_‐IFN‐β‐P2A‐NanoLuc‐pA::P_mPGK_‐ZeoR‐P2A‐EGFP‐pA‐ITR (P_RL_, 5 × UAS‐P_hCMVmin_‐Kozak) and the Sleeping Beauty transposase expression vector (P_hCMV_‐SB100X‐pA) at a ratio of 4.5:4.5:1. After selection with 1 µg mL^−1^ puromycin (Catalog no. A1113803, Thermo Fisher Scientific) and 100 µg mL^−1^ zeocin (Catalog no. R25001, Thermo Fisher Scientific) for two weeks, the surviving population was picked for further cultivation and stimulated by RL using a custom‐designed 4 × 6 light‐emitting diode (LED) array (2 mW cm^2^, 660 nm, Shenzhen Bested Opto‐electronic) with each LED centered above a single well for 24 h. NanoLuc and IFN‐β production in the culture supernatant were scored at 24 h after the initial illumination. The monoclonal iPSCs showed high sensitivity to RL (referred to as “RIVA”) were used for the following studies. All stable cell lines were regularly tested for the absence of *mycoplasma* and bacterial contamination.

### Reversibility Performance of the RID System in iPSCs and RIVA

For reversibility performance of the RID system in iPSCs, mouse iPSCs were plated into a 24‐well plate (2 × 10^4^ per well), cultivated to 70%–90% confluence, and co‐transfected with a total of 550 ng of the plasmids encoding the RID system [RL‐responsive sensor Gal4 DBD‐FnBphP (pQL326,) the RL‐dependent transactivator LDB3‐p65‐HSF1 (pNX12), and the NanoLuc reporter (pZW53) at a 3:4:4 (w/w/w) ratio]. After 24 h of transfection, the mouse iPSCs were illuminated with red light (660 nm, 0.5 mW cm^‐^^2^) for 10 s (ON) or kept in the dark (OFF). The culture medium was refreshed every 12 h with concomitant reversal of illumination conditions and NanoLuc production was quantified every 12 h for 36 h.

For reversibility performance of the RID system in RIVA, The RIVA was plated into a 24‐well plate (2 × 10^4^ per well), cultivated to 70%–90% confluence. After 24 h, the RIVA was illuminated with red light (660 nm, 0.5 mW cm^‐^^2^) for 10 s (ON) or kept in the dark (OFF). The culture medium was refreshed every 12 h with concomitant reversal of illumination conditions and IFN‐β production was quantified every 6 h for 36 h using the mouse IFN‐β ELISA Kit (Catalog no. EK2236‐96, Multi Science) according to the manufacturer's instructions.

### RL‐Controlled IFN‐β Secretion from RIVA

The RIVA was plated into a 24‐well plate (2 × 10^4^ per well), cultivated to 70%–90% confluence. After 24 h, the plate was placed below a custom‐designed 4 × 6 LED array and illuminated for different time periods (0‐18 h, 1 mW cm^‐^^2^) or light intensities (10 s, 0–2 mW cm^‐^^2^). The light intensity was determined by an optical power meter (8230E, ADC Corporation). IFN‐β production in the culture supernatant was quantified by ELISA assay (Catalog no. EK2236‐96, Multi Science) according to the manufacturer's instructions at 24 h after the initial illumination.

### Animals

BALB/c and C57BL/6 mice (female, 6–8 weeks), were ordered from East China Normal University Laboratory Animal Centre. Mice were housed in groups of 5 mice per individually ventilated cage at 22 ± 2 °C, with a 12 h light‐dark cycle and had access to food and water albidum. All animals experiment protocols were approved by the Institutional Animal Care and Use Committee of Shanghai and conducted in accordance with the National Research Council Guide for Care and Use of Laboratory Animals.

### RL‐Controlled IFN‐β Expression of RIVA In Vivo

For the preparation of mouse iPSCs or RIVA vaccines, cells were treated with DMEM containing 2 µg mL^−1^ mitomycin C (Catalog no. M5353, Sigma–Aldrich) for 5 h. Then, the cells were digested with 0.25% trypsin and made into single cell suspensions before injection.

For evaluating RL‐controlled IFN‐β expression of RIVA in a prophylactic model. Female C57BL/6 mice were subcutaneously injected with RIVA (3 × 10^6^) and illuminated with or without RL (660 nm; 20 mW cm^‐^^2^) for 10 min each day for 4 days (RIVA‐660 nm group or RIVA‐Dark group). Mice injected with wild‐type iPSCs (3 × 10^6^), or PBS were examined as controls. IFN‐β production in the prophylactic model mouse serum was quantified using a mouse IFN‐β ELISA kit at the indicated time points.

For evaluating RL‐controlled IFN‐β expression of RIVA in a therapeutic model. The A20 lymphoma mouse model was established by subcutaneous injection of 5 × 10^6^ A20 cells into the dorsum of female BALB/c mice. After the tumor volume reached 50–80 mm^3^, the mice were intratumorally injected with RIVA (3 × 10^6^), and mice were then illuminated with or without RL (20 mW cm^‐^^2^) for 10 min each day (RIVA‐660 nm group or RIVA‐Dark group). Mice injected with PBS and iPSCs (3 × 10^6^) were examined as controls. IFN‐β production in the therapeutic model mouse serum was quantified using a mouse IFN‐β ELISA kit at the indicated time points.

### Preparation and Immunization of Different Vaccines

For the preparation of the iPSCs constitutively expressing IFN‐β, iPSCs were transfected using Lipofectamine 3000‐based protocol. Briefly, 6 × 10^4^ cells per well were plated into a 6‐well plate and transfected with pNX228 (ITR‐P_EF1α_‐IFN‐β‐pA::P_mPGK_‐ZeoR‐P2A‐EGFP‐pA‐ITR, 1 µg) using Lipofectamine 3000 (Catalog no. L3000015, Thermo Fisher Scientific) according to the manufacturer's instructions. The transfected cells (3 × 10^6^) were treated with DMEM containing 2 µg mL^−1^ mitomycin C (Catalog no. M5353, Sigma‐Aldrich) for 5 h and digested into single cell suspensions.

Mice were placed in an induction chamber and anesthetized with 2% isoflurane (Catalog no. R510‐22‐10, RWD Life Science) in 100% oxygen until the loss of righting reflex. Mice were subcutaneously immunized with mitomycin C treated iPSCs (3 × 10^6^) or RIVA (3 × 10^6^) or iPSCs constitutively expressing IFN‐β three times at 4‐day intervals.

### In Vivo Prophylactic and Therapeutic Tumor Model Experiments

For the therapeutic tumor vaccination studies, the female BALB/c mice were implanted with A20 cells (5 × 10^6^) or CT26 colon cancer cells (1 × 10^6^) under the dorsum skin of mice. After the tumor average volume reached 50 – 80 mm^3^, the mice were subcutaneously injected with RIVA (3 × 10^6^) three times at 4‐day intervals, and mice were then illuminated with or without RL (20 mW cm^‐^^2^) for 10 min each day for 12 days (RIVA‐660 nm group or RIVA‐Dark group). The examined controls included mice injected with wild‐type iPSCs (3 × 10^6^), or IFN‐β (200 ng per mouse, Catalog no. 50708‐MCCH, Sino Biological), or iPSCs + IFN‐β or PBS.

For the prophylactic tumor vaccination studies, the female BALB/c mice were subcutaneously injected with RIVA (3 × 10^6^) three times at 4‐day intervals, and mice were then illuminated with or without RL (20 mW cm^‐^^2^) for 10 min each day for 12 days (RIVA‐660 nm group or RIVA‐Dark group). The examined controls included mice injected with wild‐type iPSCs (3 × 10^6^), or IFN‐β (200 ng per mouse), or iPSCs + IFN‐β or PBS. After 12 days, the immunized mice were subcutaneously implanted with A20 (2.5 × 10^6^) or B16F10_OVA_ (1 × 10^5^) or 4T1_luc_ (1 × 10^6^) cells.

The tumor volumes were measured every three days by a digital caliper and calculated by the following formula: Tumor volume = (length × width^2^) / 2. The bioluminescence signal of lungs metastasis 4T1_luc_ was measured every three days using an IVIS Lumina II in vivo imaging system (Perkin Elmer). Animals were euthanized with carbon dioxide asphyxiation when a tumor size greater than 2000 mm^3^.

### Tumor Re‐Challenge

To establish the A20 tumor rechallenge model, the mice vaccinated with the RIVA (RIVA‐660 nm group and remained tumor free after 60 days were rechallenged by subcutaneous injection of A20 cells (2.5 × 10^6^) or CT26 colon cancer cells (1 × 10^6^) on the contralateral flank alongside naïve age‐matched controls. The volumes of all tumors were measured with a digital caliper every 3 days.

### In Vivo Bioluminescence Assay

In the 4T1_luc_ pulmonary metastasis model, mice bearing 4T1_luc_ breast tumor cells were intraperitoneally injected with 15 mg mL^−1^ D‐Luciferin potassium solution (150 mg kg^−1^; Catalog no. luc001, Shanghai Scielight Biology Science & Technology) and anesthetized with 2% isoflurane (Catalog no. R510‐22‐10, RWD Life Science) dissolved in oxygen using an Economical animal anesthesia machine (HSIV‐S, Raymain). After 10 min injection, bioluminescence images of the mice were obtained by an IVIS Lumina II in vivo imaging system (Perkin Elmer) and the total flux of the tumor was analyzed with Living Image software (version 4.3.1).

### Isolation of Peripheral Blood Lymphocytes

Peripheral blood samples were collected from mouse orbit and transferred to a blood collection tube coated with an ethylenediaminetetraacetic acid (EDTA) anticoagulant (Catalog no. BD‐68784, BD Biosciences), then immediately isolated in a centrifuge at 1000 × *g* for 10 min and plasma was discarded. The blood sample was lysed in 1 × red blood cell (RBC) lysis buffer (Catalog no. C3702, Beyotime Biotechnology) for 10 min at room temperature (RT). Subsequently, the samples were centrifuged at 1000 × *g* for 10 min to remove the lysed RBCs in the supernatant and the pellets containing lymphocytes were washed with FACS buffer (90% PBS + 10% FBS) and resuspended in a mouse CD16/32 antibody solution (Catalog no. 156 604, Biolegend) to block non‐specific and FcR‐mediated antibody binding.

### Isolation of Spleen and Lymphatic Node Lymphocytes

Spleen and lymphatic node cell suspensions were obtained by grinding the tissue through a 70 µm cell strainer (Catalog no. 93 070, SPL Life Sciences). The dissociated cells were treated with RBC lysis buffer (Catalog no. C3702, Beyotime Biotechnology) for 10 min at room temperature. Subsequently, the samples were centrifuged at 1000 × *g* for 10 min to remove the lysed RBCs in the supernatant and the pellets containing the spleen lymphocytes were washed with FACS buffer (90% PBS + 10% FBS) and resuspended in a mouse CD16/32 antibody solution (Catalog no. 156 604, Biolegend) to block non‐specific and FcR‐mediated antibody binding.

### Flow Cytometry

To evaluate which immune cells are required to confer the observed anti‐tumor effect, the single cell suspension specific cell subsets (CD86^+^MHCII^+^ DC cells, CD69^+^ NK cells, CD4^+^ T cells, CD8^+^ T cells, CD69^+^CD8^+^ T cells, IFN‐γ^+^CD8^+^ T cells) were stained with fluorescence‐labelled antibodies, then analyzed using a flow cytometry. Single‐cell suspensions were stained using a Zombie NIR Fixable Viability Kit (Catalog no. 423 105, Biolegend) for 20 min. The antibodies used for marking DC cells were CD45‐Brilliant Violet 421 (Catalog no. 103 134, Biolegend), CD11c‐FITC (Catalog no. 117 306, Biolegend), CD86‐PE (Catalog no. 105 007, Biolegend), and I‐A/I‐E‐PE/Cyanine7 (Catalog no. 107 629, Biolegend). The antibodies used for marking CD4^+^ cells were CD45‐Brilliant Violet 421 (Catalog no. 103 134, Biolegend), CD3‐FITC (Catalog no. 100 306, Biolegend), and CD4‐PerCP/Cyanine5.5 (Catalog no. 100 434, Biolegend). The antibodies used for marking CD8^+^ cells were CD45‐Brilliant Violet 421 (Catalog no. 103 134, Biolegend), CD3‐FITC (Catalog no. 100 306, Biolegend), and CD8α‐APC (Catalog no. 100 712, Biolegend). The antibodies used for marking CD69^+^CD8^+^ T cells were CD45‐Brilliant Violet 421 (Catalog no. 103 134, Biolegend), CD3‐FITC (Catalog no. 100 306, Biolegend), CD8α‐APC (Catalog no. 100 712, and CD69‐PE/Cyanine7 (Catalog no. 104 512, Biolegend). The antibodies used for marking CD69^+^CD49b^+^ cells were CD45‐Brilliant Violet 421 (Catalog no. 103 134, Biolegend), CD3‐FITC (Catalog no. 100 306, Biolegend), CD49b‐PE (Catalog no. 108 908, Biolegend), and CD69‐PE/Cyanine7 (Catalog no. 104 512, Biolegend).

To analyze the intracellular IFN‐γ positive T cells in the spleen, splenocytes collected at the end of the experiment were incubated with Cell Stimulation Cocktail stimulating (Catalog no. 2 524 260, Thermo Fisher Scientific) for 6 h at 37 °C in culture medium containing RPMI 1640 supplemented with 10% FBS. Next, cells were washed in PBS buffer and stained with CD45‐Brilliant Violet 421 (Catalog no. 103 134, Biolegend), CD3‐FITC (Catalog no. 100 306, Biolegend), and CD8α‐APC (Catalog no. 100 712, Biolegend) for 30 min on ice. Then, cells were fixed and permeabilized with Intracellular Staining Permeabilization Wash Buffer (Catalog no. 421 002, Biolegend) according to the manufacturer's protocol. Finally, cells were stained with IFN‐γ‐PerCP/Cyanine5.5 (Catalog no. 505 822, Biolegend) for 30 min on ice, washed in PBS buffer and analyzed using a BD LSRFortessa Flow Cytometer (BD Biosciences). A minimum of 5000 events per plot were collected and analyzed using the FlowJo V10.6.2 software. The numbers presented in the flow cytometry analysis images are percentage based.

### Depletion of NK, CD4^+^ T and CD8^+^ T Cells

Female BALB/c mice were subcutaneously inoculated with RIVA (3 × 10^6^ per mouse). Cellular subsets were depleted by intraperitoneally injecting depleting antibodies (200 µg mouse^−1^) three times at 4‐day intervals. After 12 days, the immunized mice were subcutaneously injected with 5 × 10^6^ A20 cells and intraperitoneally injected with NK, CD4 or CD8α depleting antibodies (200 µg mouse^−1^) every two weeks for three times in total. The antibodies used for depletion were NK (Catalog no. 103 525; Biolegend), CD4 (Catalog no. 100 442; Biolegend) or CD8α (Catalog no. 100 764; Biolegend).

### Serum Cytokines Detection

Serum cytokines (IFN‐β, TNF‐α, and IL‐6) were measured using corresponding enzyme‐linked immunosorbent assay (ELISA) kits according to the manufacturer's instructions. The used ELISA kits were IFN‐β, (Catalog no. EK2236‐96, Multi Science), TNF‐α (Catalog no. EK282, Multi Science), and IL‐6 (Catalog no. EK206, Multi Science).

### Hematoxylin and Eosin (H&E) Staining

Samples from the heart, liver, spleen, lung, and kidney tissues were collected and fixed in 4% paraformaldehyde (Catalog no. G1101, Servicebio) overnight at room temperature. The fixed samples were gently dehydrated by immersing them in a graded series of ethyl alcohol solutions, cleaned in xylene, embedded in paraffin, and sliced into 4 µm‐thick sections with a rotary microtome (Leica RM2235, Manual Rotary Microtome). These sections were stained with an H&E Staining Kit (Catalog no. G1005, Servicebio) following the manufacturer's instruction and observed under a BX53 upright fluorescence microscope (Olympus).

### Complete Blood Count and Liver/Kidney Function Analysis

Mice were euthanized and whole blood was collected and immediately analyzed for complete blood count using the Sysmex XT‐2000iV hematology analyzer (Sysmex). The parameters of hepatic function including alanine aminotransferase (ALT), aspartate aminotransferase (AST), and albumin/globulin ratio (A/G) as well as the parameters of kidney function including creatinine (CRE) and blood urea nitrogen (BUN) were measured using an automatic biochemical analyzer BX‐3010 (Sysmex). All biochemical serum evaluations were performed at the same time to minimize analytical variability.

### Statistical Analysis

Unless otherwise mentioned, all in vitro data represent means ± SD of three independent biological replicates. For the animal experiments, each treatment group consisted of randomly selected mice (*n* = 5–12) and the results are expressed as means ± SEM. Comparisons between two groups were performed using two‐tailed unpaired Student's *t*‐test. A one‐way ANOVA with Tukey multiple hypothesis correction was used to assess significance between more than two groups, and two‐way ANOVA was used when comparing across two factors and adjusted for multiple hypothesis correction by Sidak's multiple comparisons test. Survival data were analyzed using the log‐rank (Mantel‐Cox) test for multiple comparisons. GraphPad Prism 8 software was used for statistical analysis and statistical significance was set as follows: *P < 0.05, **P < 0.01, ***P < 0.001, and ****P < 0.0001, and ns denotes no significant difference. *n* and *P* values were described in the figure legends.

## Conflict of Interest

The authors declare no conflict of interest.

## Author Contributions

L.Q., L.N., Z.W. contributed equally to this work. H.Y., M.W. and F.C. conceived the project. H.Y., L.Q., and M.W. designed the experiments, analyzed the results, and wrote the manuscript. L.Q., L.N., Z.W., M.W., D.D., S.T., X.M., Z.D., G.Y., Y.Z., T.Y., X.L., D.K., L.H., X.L., and J.Z. performed the experimental work. L.Q., M.W., L.N., and Z.W. designed, analyzed, and interpreted the experiments. All authors edited and approved the manuscript.

## Supporting information



Supporting Information

## Data Availability

All data associated with this study are present in the paper or the Supplementary Information. All genetic components related to this paper are available with a material transfer agreement and can be requested from H.Y. (hfye@bio.ecnu.edu.cn).
