# Supplementary material for: Optogenetic‐Controlled iPSC‐Based Vaccines for Prophylactic and Therapeutic Tumor Suppression in Mice
Source: Adv Sci (Weinh). 2025 Jul 6;12(36):e16115. doi: 10.1002/advs.202416115 (PMC12463003; doi:10.1002/advs.202416115)
Supplement: Supplementary file 1 — Supporting Information [file ADVS-12-e16115-s001.docx]

**Supplementary Information**

**Optogenetic-controlled iPSC-based vaccines for prophylactic and therapeutic tumor suppression in mice**

Longliang Qiao, Lingxue Niu, Zhihao Wang, Di Dai, Shasha Tang, Xiaoding Ma, Zhenqiang Deng, Guiling Yu, Yang Zhou, Tao Yan, Xingwan Liu, Deqiang Kong, Linfeng Hu, Xiang Li, Junwei Zhao, Fengfeng Cai^*^, Meiyan Wang*, Haifeng Ye*


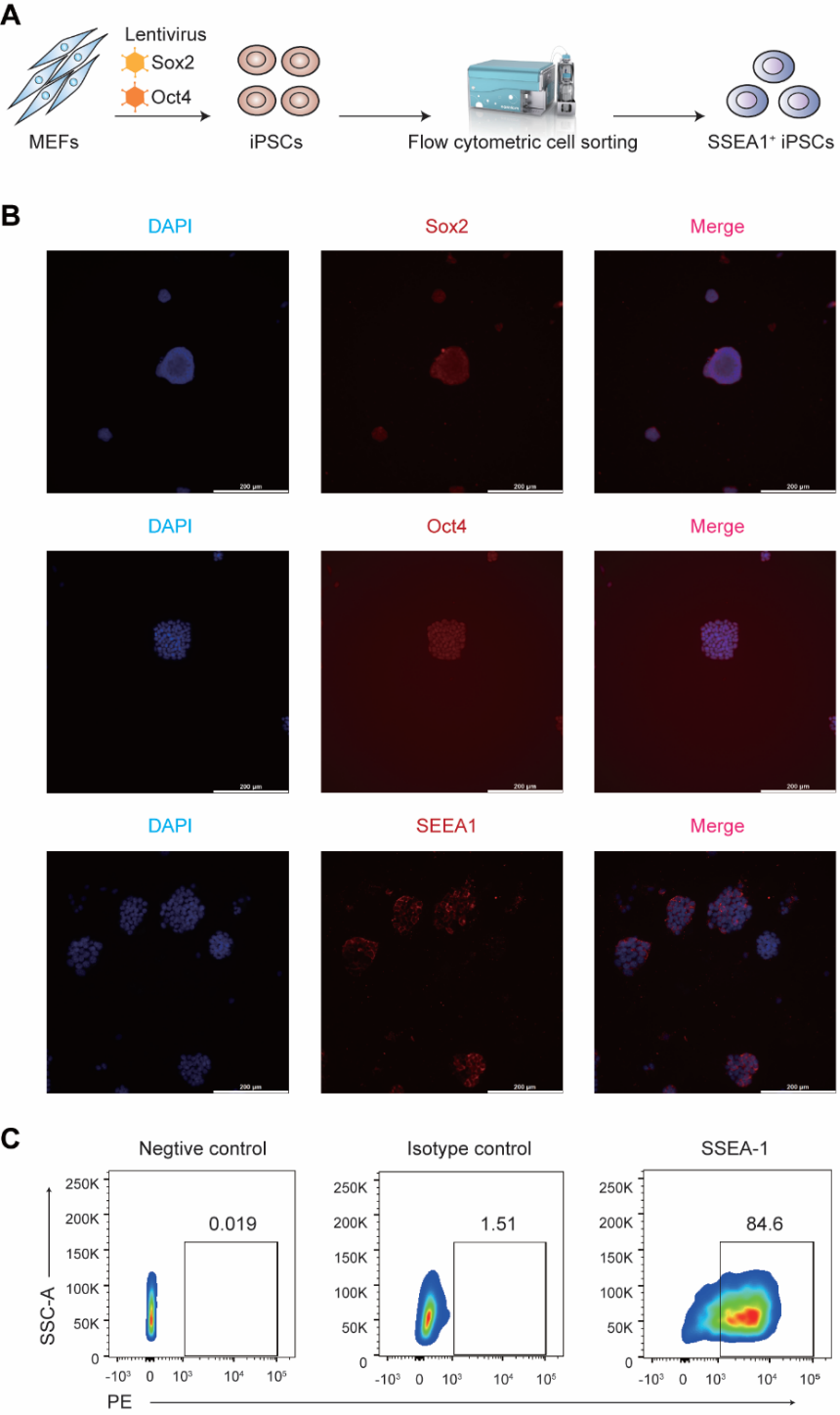


**Fig. S1. iPSCs derived from mouse embryonic fibroblasts (MEFs).** (**A**) Schematic showing the experimental procedure and the time schedule used for reprogramming of mouse MEFs into iPSCs. (**B**) Representative fluorescence microscopy images of pluripotency markers including Sox2 (Red), Oct4 (Red), and SSEA-1 (Red) in the obtained iPSCs. Blue indicates DAPI staining of nuclei. Scale bar, 200 µm. (**C**) Representative flow cytometry staining of SSEA1^+^ iPSCs.


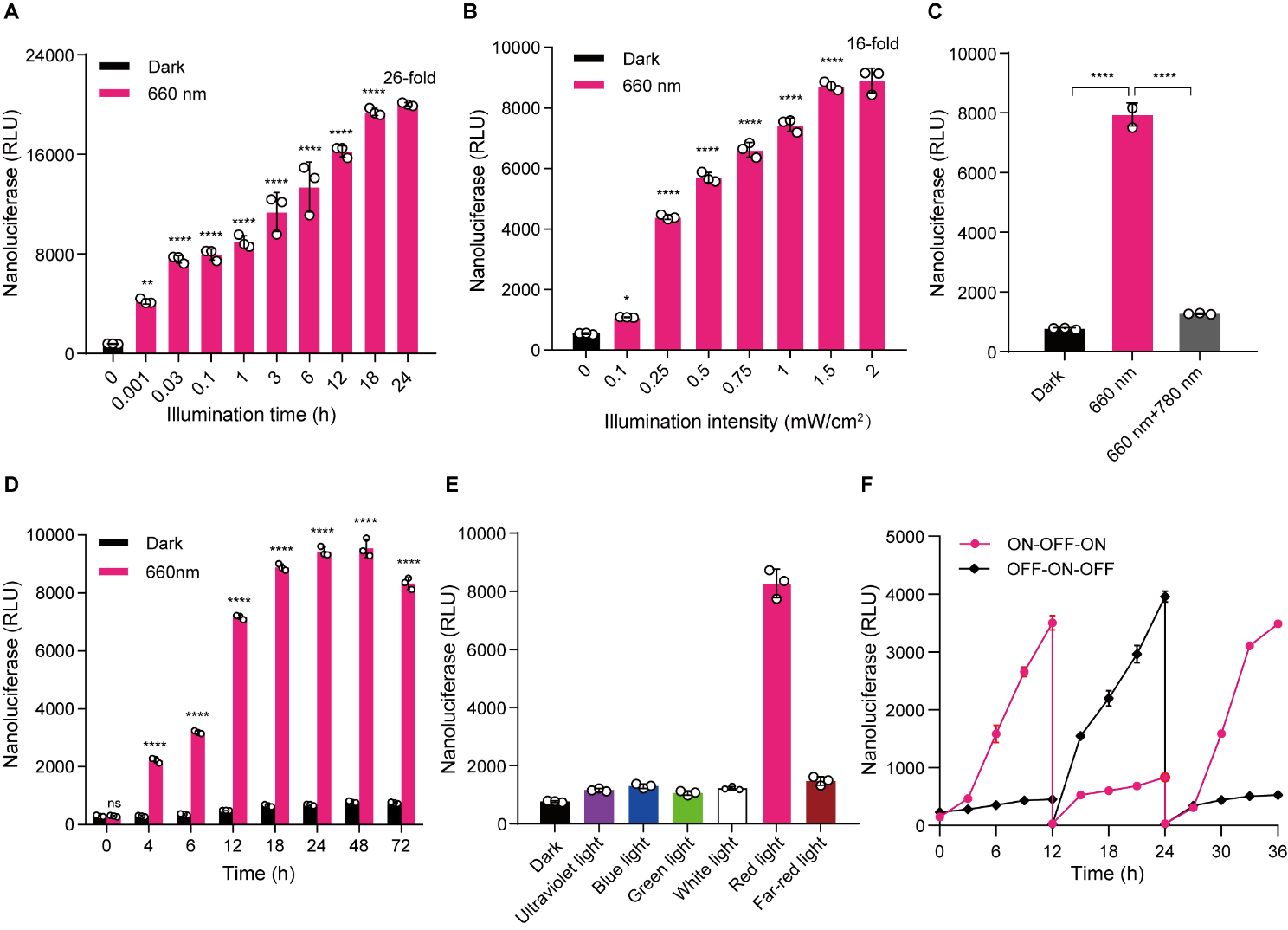


**Fig. S2. Characterization of the RID system in iPSCs.** (**A**) Assessment of illumination time-dependent RID-mediated transgene expression kinetics. iPSCs (2 × 10^4^) were co-transfected with Gal4-FnBphP vector (pQL326), 2NLS-LDB3-p65-HSF1 expression vector (pNX12), and NanoLuc reporter expression vector (pZW53) at a 3:4:4 (w/w/w) ratio, and then illuminated with RL (660 nm, 1.0 mW/cm^2^) for the indicated time period (0-24 h). NanoLuc production was quantified at 24 h after the initial illumination. (**B**) Illumination intensity-dependent RID-mediated transgene expression kinetics. iPSCs (2 × 10^4^) transfected as described in (A) were illuminated with RL (660 nm) at the indicated light intensities (0-2.0 mW/cm^2^) for 10 s. NanoLuc production was quantified as described in (A). (**C**) The switch ON/OFF performance of the RID system. Three groups of iPSCs were co-transfected as described in (A). 24 h after transfection, cells were illuminated with RL for 10 s and switched to the dark condition or 780 nm illumination at 1.0 mW/cm^2^ for 2 min for the “660 nm+780 nm” group. As a control, cells were kept in the dark throughout the experiment. NanoLuc production was quantified at 24 h after 660 nm or 780 nm illumination. (**D**) Quantification of RID-mediated transgene expression kinetics. iPSCs (2 × 10^4^) transfected as described in (A) were illuminated with RL (660 nm, 1.0 mW/cm^2^) for 10 s. NanoLuc production in the culture supernatant was profiled at the indicated time period (0 - 72 h) after illumination. (**E**) Chromatic specificity of the RID system. iPSCs (2 × 10^4^) transfected as described in (A) were illuminated with the indicated wavelengths of light at 1.0 mW/cm^2^ for 10 s. NanoLuc production was quantified 24 h after illumination. (**F**) Reversibility of RID-mediated transgene expression. iPSCs (2 × 10^4^) transfected as described in a were illuminated with RL (660 nm, 0.5 mW/cm^2^) for 10 s (ON) or kept in the dark (OFF). NanoLuc production was quantified every 12 h for 72 h; the culture medium was renewed every 12 h. Data in (A to F) are presented as means ± SD; *n* = 3 independent experiments. *P* values in (A and B) were calculated by one-way ANOVA with a Turkey’s test. *P* values in (C and D) were calculated by two-tailed unpaired Student’s *t*-test. ns, not significant, **P* < 0.05, ***P* < 0.01, *****P* < 0.0001. Detailed descriptions of the genetic constructs and transfection mixtures are provided in **Tables S1** and **S2**.


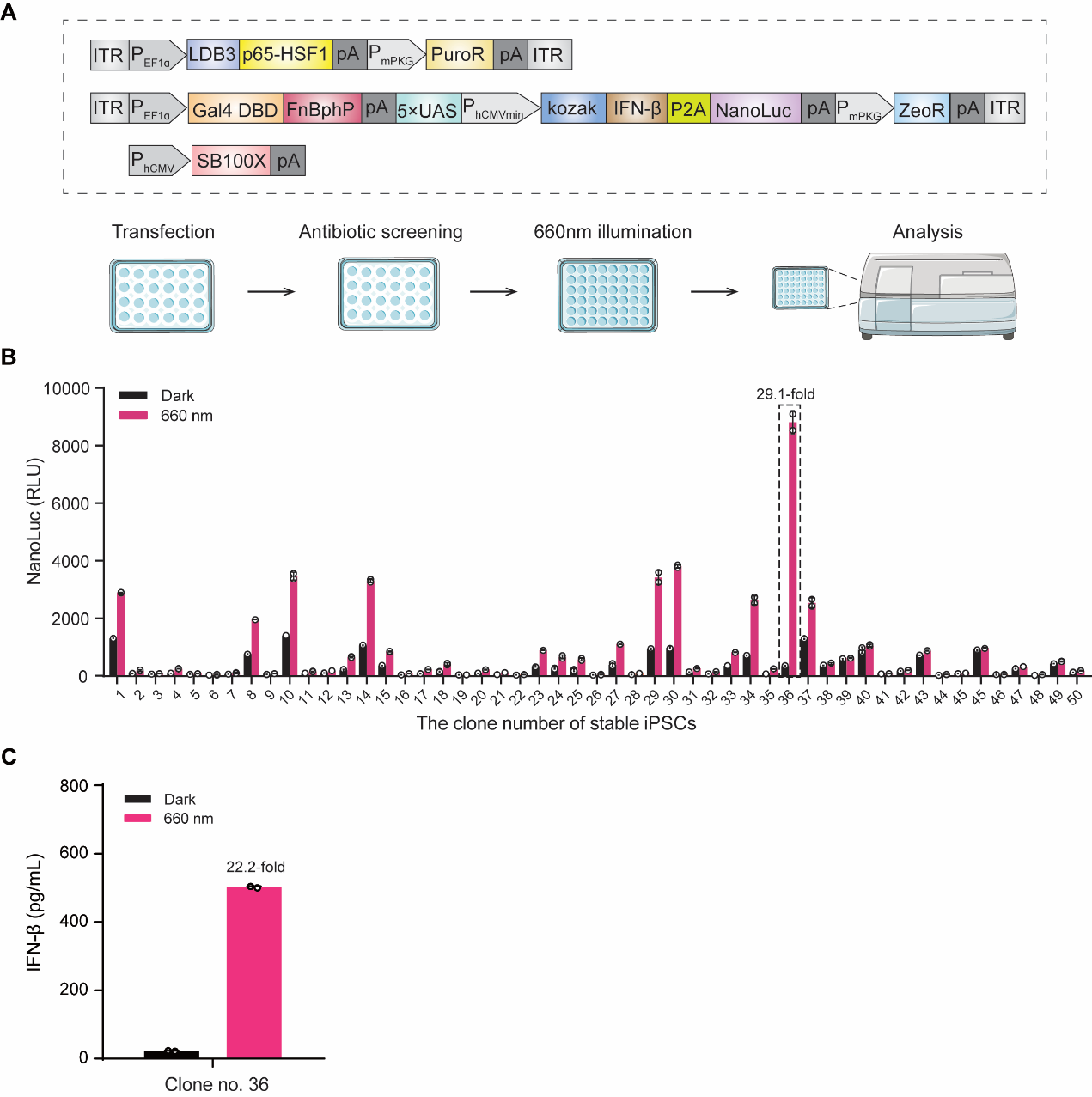


**Fig. S3.** **Selection of stable red/far-red light-controlled iPSC-based vaccine (RIVA).** (**A**) Schematic showing the experimental procedure for transfecting and establishing stable RIVA using the Sleeping Beauty transposon system. Mouse iPSCs were stably integrated with pNX176 (P_EF1α_-Gal4 DBD-FnBphP-pA::P_RL_-IFN-β-P2A-Nanoluc-pA::P_mPGK_-ZeoR-P2A-EGFP-pA) and pNX175 (P_EF1α_-2NLS-LDB3-p65-HSF1-pA::P_mPGK_-PuroR-pA), and selected with 1 μg/mL puromycin and 100 μg/mL zeocin for two weeks, the surviving population was picked for further analysis. (**B**) Fifty randomly selected cell clones were profiled for their RL-inducible Nanoluc production. (**C**) Analysis of RL-inducible cytokine IFN-β production for clone no. 36.


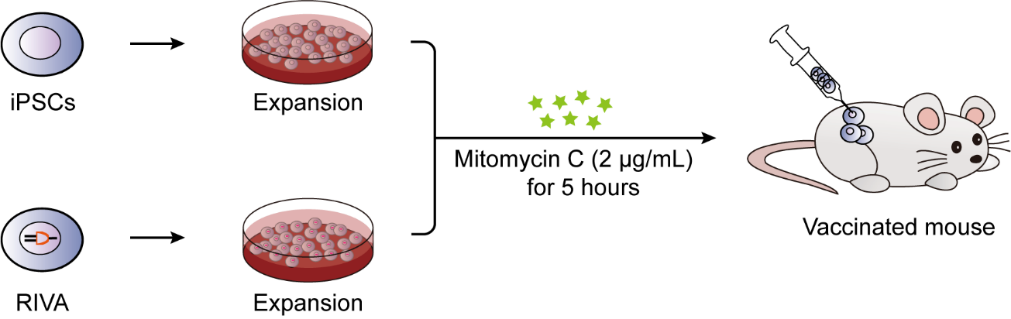


**Fig. S4. Mitomycin C treated iPSCs and RIVA before subcutaneous injection to mice.** Schematic showing the experimental procedure for preparation of iPSCs and RIVA used for vaccinating mice. iPSCs and RIVA were treated with 2 µg/mL mitomycin C for 5 h to suppress cell proliferation, and then subcutaneously injected into mice.


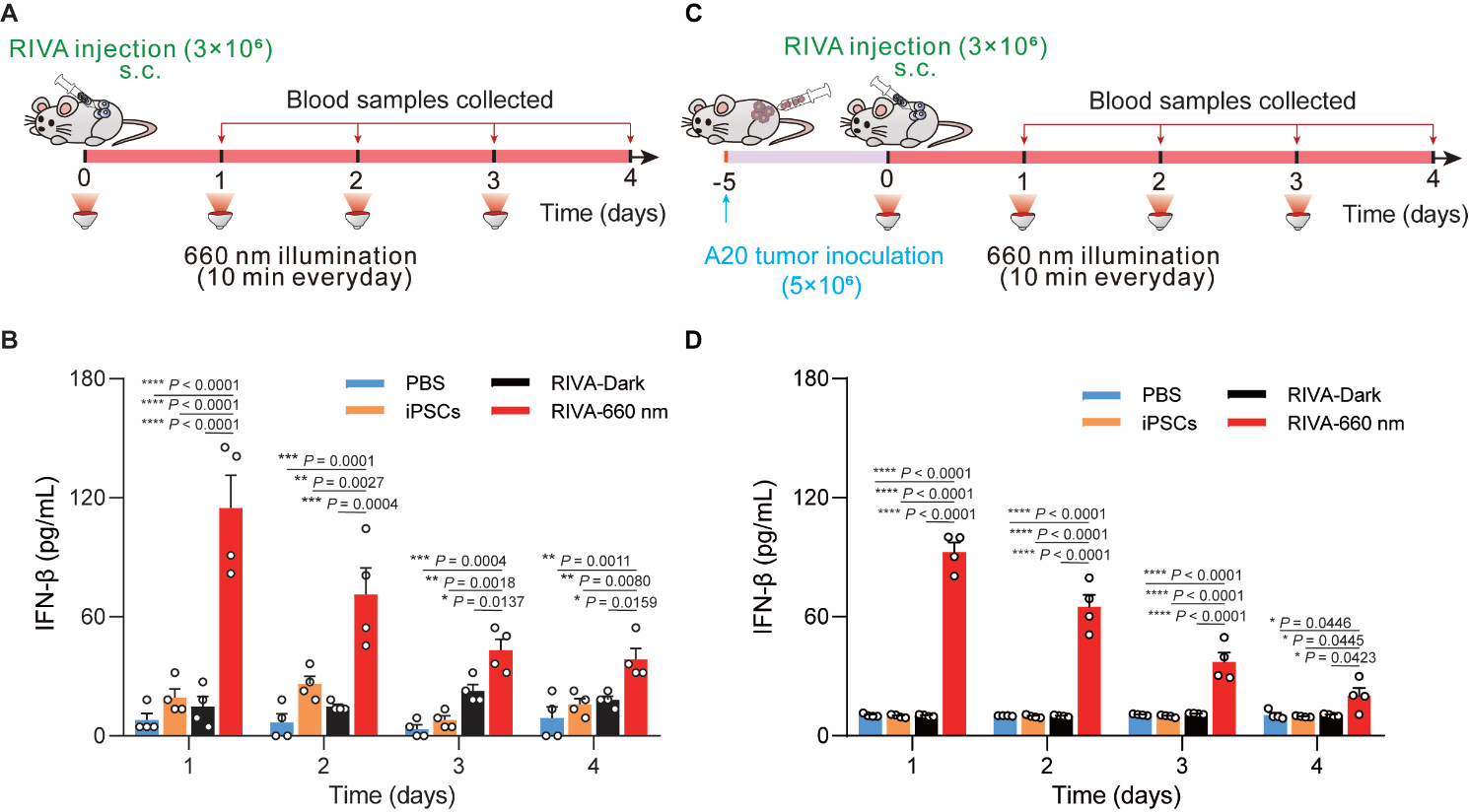


**Fig. S5.** **RL-controlled IFN-β expression from RIVA in vivo.** **(A)** Schematic representation of the experimental procedure for assessing RL-controlled IFN-β expression by RIVA in a prophylactic mouse model. Female C57BL/6 mice were subcutaneously injected with RIVA cells (3 × 10⁶), followed by daily illumination with red light (RL, 660 nm; 20 mW/cm²) or no illumination for 10 min each day over 4 consecutive days (RIVA-660 nm or RIVA-Dark groups, respectively). Control mice were injected with wild-type iPSCs (3 × 10⁶) or PBS. **(B)** IFN-β production in serum samples collected from prophylactic model mice was quantified at indicated time points using a mouse IFN-β enzyme-linked immunosorbent assay (ELISA) kit. **(C)** Schematic representation of the experimental procedure for evaluating RL-controlled IFN-β expression by RIVA in a therapeutic mouse model. The therapeutic model was established by subcutaneous injection of 5 × 10⁶ A20 lymphoma cells into the dorsal region of female BALB/c mice. Once tumor volumes reached 50–80 mm³, mice were intratumorally injected with RIVA cells (3 × 10⁶) and subsequently illuminated daily with or without RL (660 nm; 20 mW/cm²) for 10 min each day (RIVA-660 nm or RIVA-Dark groups, respectively). Control mice received injections of PBS or wild-type iPSCs (3 × 10⁶). **(D)** IFN-β production in serum samples collected from therapeutic model mice was quantified at indicated time points using a mouse IFN-β ELISA kit. Data in panels (B) and (D) are presented as means ± SEM (*n* = 4 mice per group). Statistical significance in (B) and (D) was determined by one-way ANOVA with Tukey’s test. **P* < 0.05, ***P* < 0.01, ****P* < 0.001, *****P* < 0.0001.


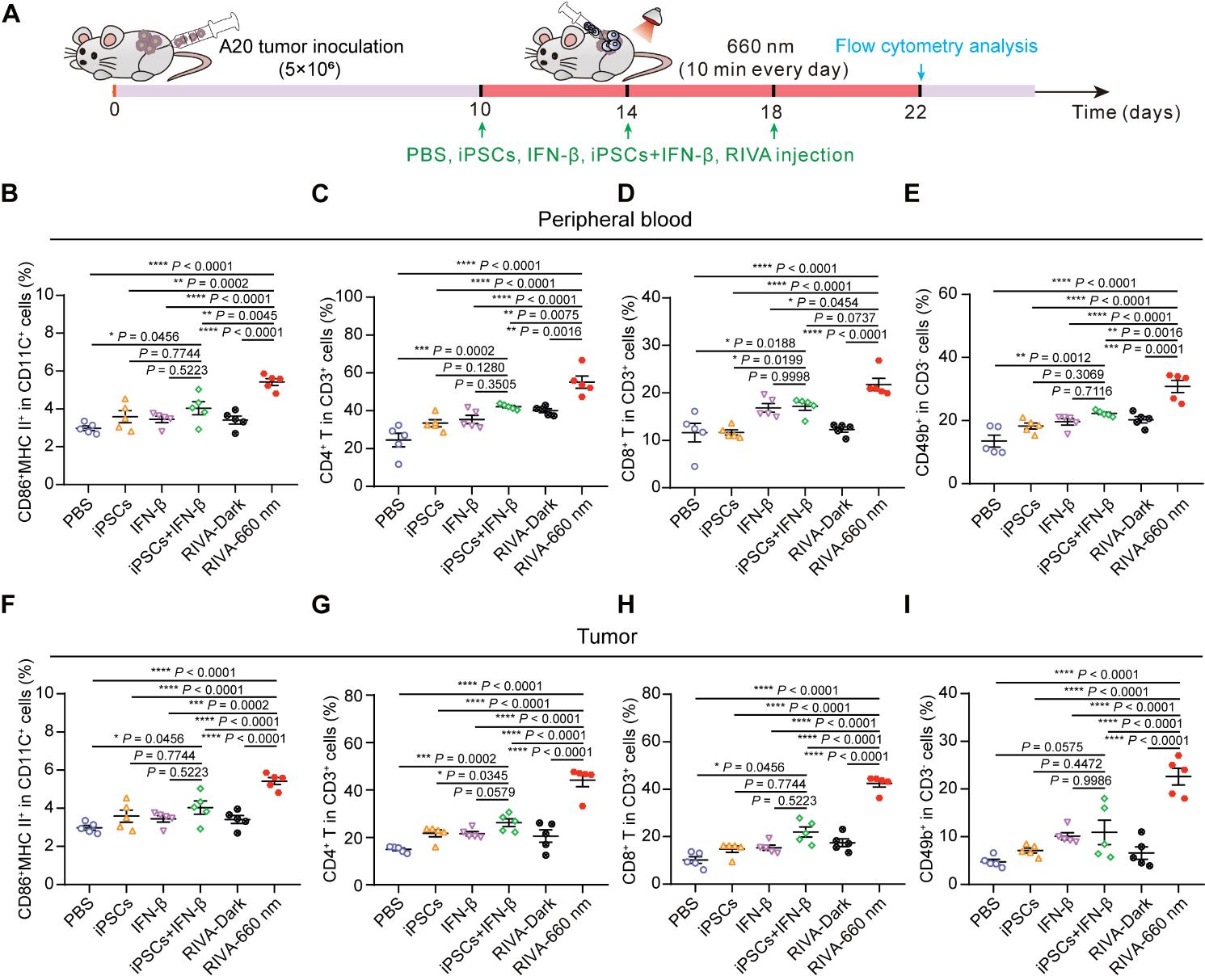


**Fig. S6.** **Anti-tumor immune responses elicited by RIVA in a therapeutic model.** **(A)** Schematic representation of the experimental procedure used to evaluate antitumor immune responses induced by therapeutic RIVA in an A20 lymphoma mouse model. The model was established by subcutaneously injecting 5 × 10⁶ A20 cells into the dorsal region of female BALB/c mice. When tumor volumes reached approximately 300 mm³, mice were intratumorally injected with RIVA cells (3 × 10⁶) three times at 4-day intervals, followed by daily illumination with or without RL (660 nm; 20 mW/cm²) for 10 min each day over a period of 12 days (designated as RIVA-660 nm or RIVA-Dark groups, respectively). Control groups included mice injected with wild-type iPSCs (3 × 10⁶), IFN-β alone (200 ng per mouse), a combination of iPSCs and IFN-β (iPSCs + IFN-β), or PBS. Twelve days after the different treatments, the proportions of mature DCs (CD86⁺MHC II⁺), CD4⁺ T cells, CD8⁺ T cells, and NK cells (CD49b⁺) in peripheral blood (B–E) and tumor tissues (F–I) were analyzed by flow cytometry. Data in panels B–I represent means ± SEM (*n* = 5 mice per group). Statistical significance in panels B–I was determined using one-way ANOVA with Tukey’s test. **P* < 0.05, ***P* < 0.01, ****P* < 0.001, *****P* < 0.0001.


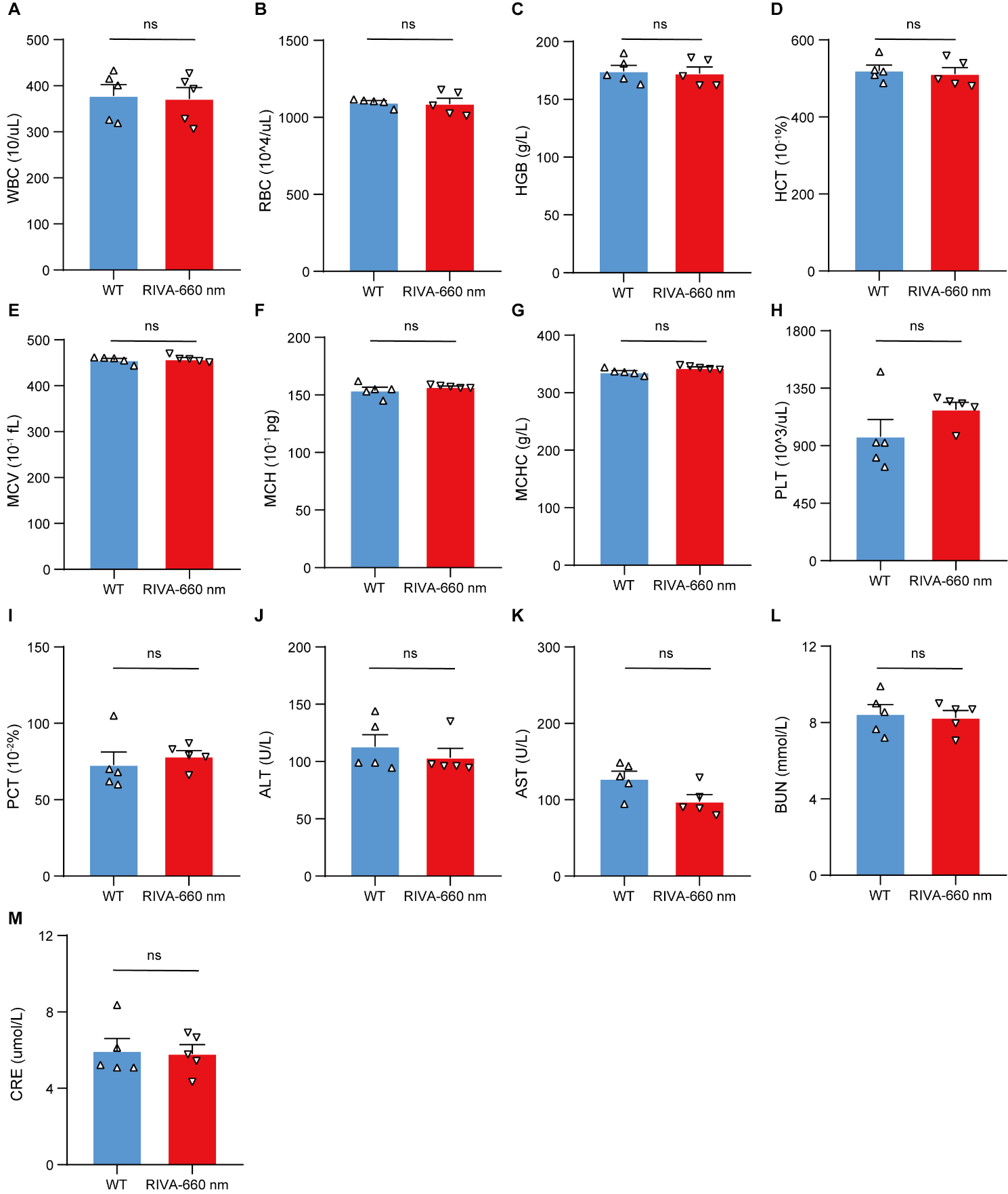


**Fig. S7. Blood biochemistry and hematology analysis of BALB/c mice injected with RIVA under RL illumination and healthy wild-type (WT) BALB/c control mice.** Mouse blood was collected on day 100 from untreated healthy mice and the rechallenged mice. (**A**) WBC (white blood cells), (**B**) RBC (red blood cells), (**C**) HGB (hemoglobin), (**D**) HCT (hematocrit), (**E**) MCV (mean corpuscular volume), (**F**) MCH (mean corpuscular hemoglobin), (**G**) MCHC (mean corpuscular hemoglobin concentration), (**H**) PLT (platelets) and (**I**) PCT (platelet crit) were tested using Sysmex XT-2000i automated hematology analyzer. (**J**) ALT (alanine aminotransferase), (**K**) AST (aspartate aminotransferase), (**L**) BUN (blood urea nitrogen) and (**M**) CRE (creatinine) were tested using an automatic biochemical analyzer BX-3010 (Sysmex). All data are presented as means ± SEM (*n* = 5 mice per group). *P* values were calculated by two-tailed unpaired Student’s *t*-test. ns, not significant.


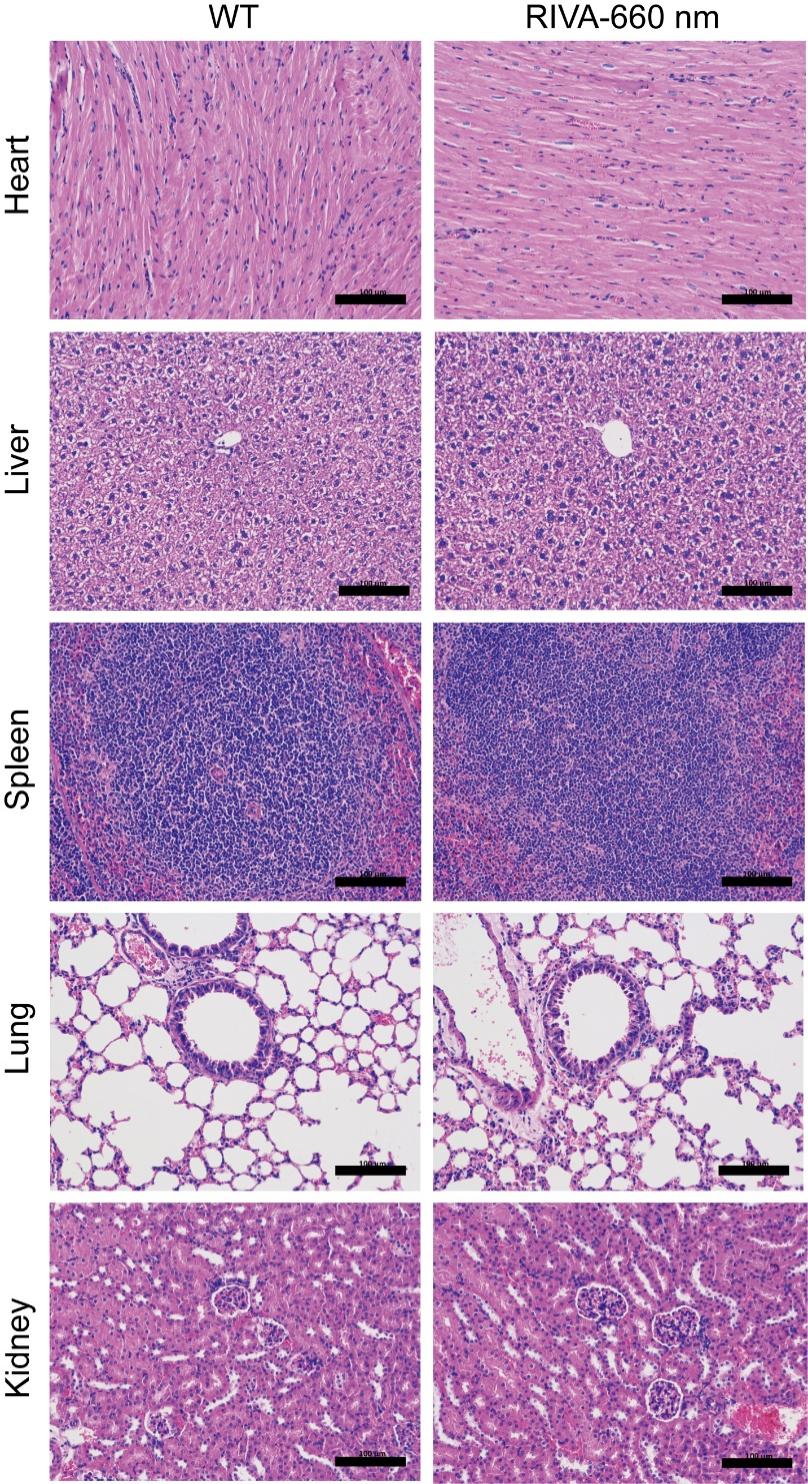


**Fig. S8. Representative H&E staining of major organs of BALB/c mice injected with RIVA under RL illumination and healthy wild-type (WT) BALB/c control mice.** Mouse major organs including heart, liver, spleen, lung, and kidney were collected on day 100 from untreated healthy mice and the rechallenged mice and stained by H&E. Scale bar, 100 µm.


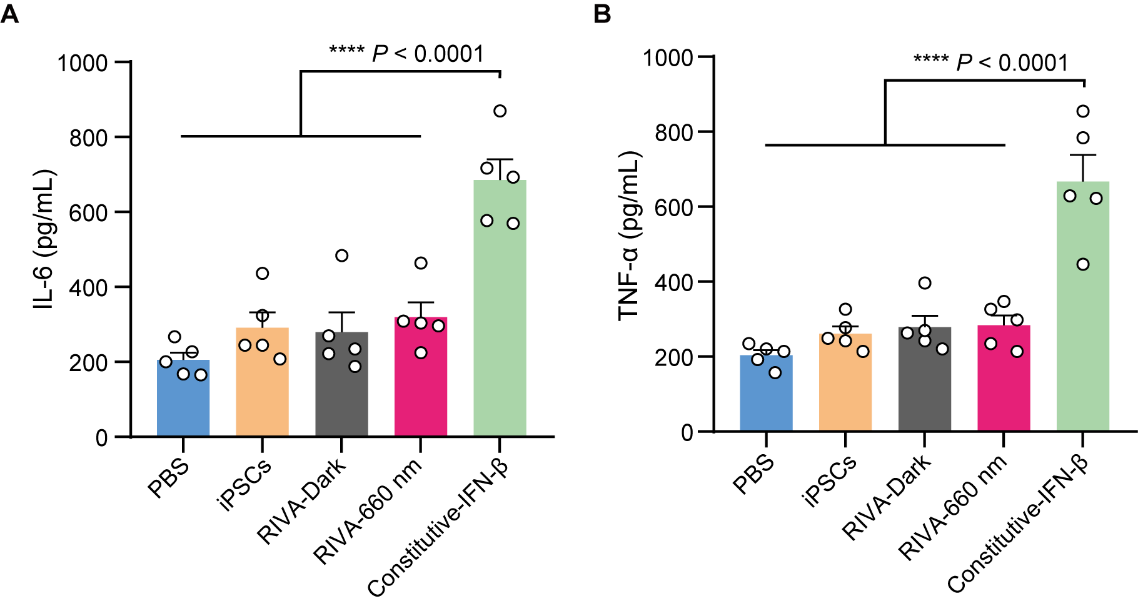


**Fig. S9. Serum inflammatory cytokines in different mouse groups.** BALB/c mice were subcutaneously injected with RIVA (3 × 10^6^) three times at 4-day intervals, and mice were then illuminated with or without RL (660 nm; 20 mW/cm^2^) for 10 min (RIVA-660 nm group or RIVA-Dark group). Mice injected with iPSCs constitutively expressing IFN-β (3 × 10^6^), or wild-type iPSCs (3 × 10^6^), or PBS were examined as controls. Mouse serum was obtained 24 hours after illumination for cytokine production analysis. Cytokines (**A**) IL-6 and (**B**) TNF-α were measured using corresponding ELISA kit. Data are means ± SEM (*n* = 5 mice per group). *P* values were calculated by one-way ANOVA with a Turkey’s test. *****P* < 0.0001.


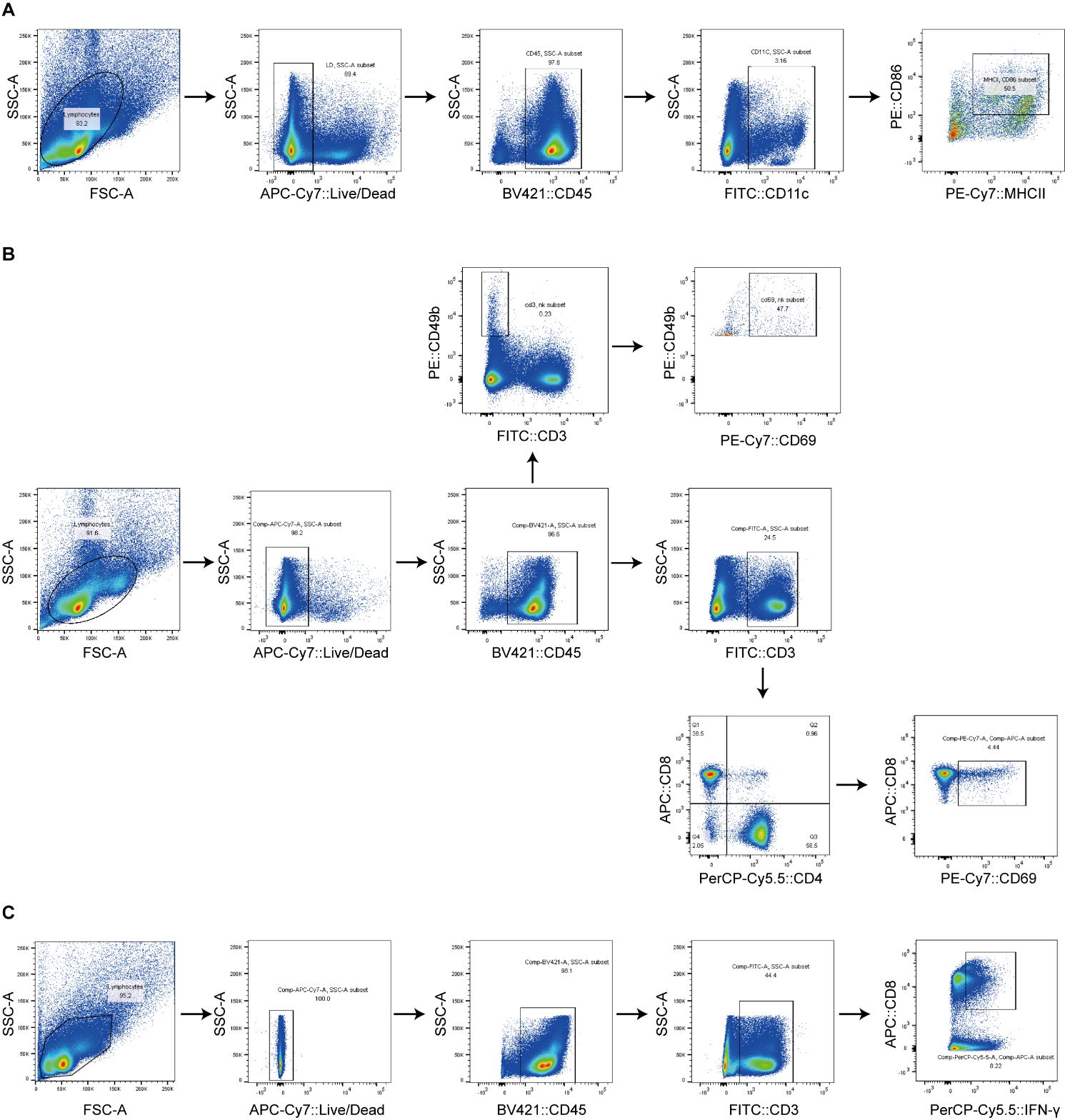


**Fig. S10.** **Gating strategies of representative flow cytometry staining of DC cells, NK cells and T cells.** DC cells, NK cells and T cells were gated on viability-dye negative cells to exclude dead cells, lymph gated on size to include lymphocytes, and doublets were excluded based on size (FSC) and granularity (SSC). Representative results of one independent experiment. (**A**) Expression of CD11c^+^MHCII^+^CD86^+^ was used to define matured DC cells. (**B**) Expression of CD45^+^CD3^+^CD8^+^ was used to define total CD8^+^ T cells. Expression of CD69^+^ and CD49b^+^ were used to define activated NK cells. (**C**) Expression of IFN-γ^+^ CD8^+^ was used to define cytotoxic CD8^+^ T cells.


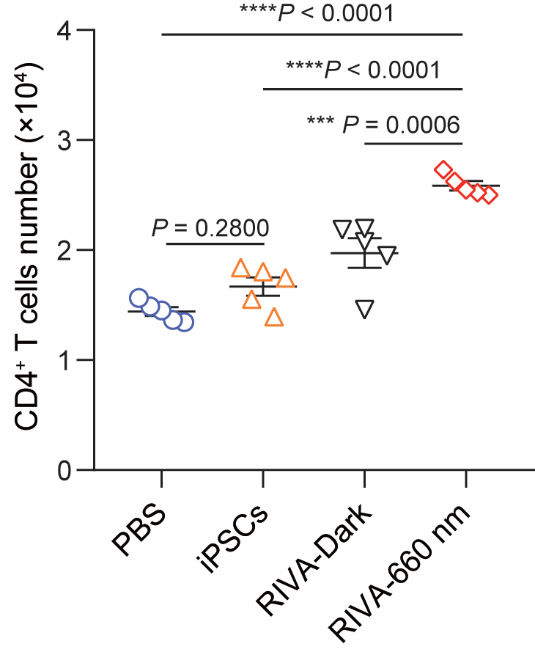


**Fig. S11.** **Numbers of CD4^+^ cells in CD45^+^ cells evaluated in the spleen of mice.** BALB/c mice were subcutaneously injected with RIVA (3 × 10^6^) three times at 4-day intervals and illuminated with or without RL (20 mW/cm^2^) for 10 min each day for 12 days (RIVA-660 nm group or RIVA-Dark group). Mice injected with wild-type iPSCs (3 × 10^6^) or PBS were examined as controls. After 12 days, the immunized mice were subcutaneously injected with 5 × 10^6^ A20 cells. Spleens were removed and ground to collect lymphocytes on day 7 after injection of A20 cells. Quantification of total numbers of CD4^+^ T cells in CD45^+^ cells (1.3 × 10^5^) by flow cytometry analysis. Data are means ± SEM (*n* = 5 mice per group). *P* values were calculated by two-way ANOVA with a Sidak’s test. ****P* < 0.001, *****P* < 0.0001.

**Table S1.** Plasmids designed and used in this study.

| **Plasmid** | **Description and cloning strategy** | **Reference** |
| --- | --- | --- |
| pcDNA3.1(+) | Constitutive mammalian P_hCMV_-driven expression vector (P_hCMV_-MCS-pA) | Invitrogen’ CA |
| SB100X | Constitutive P_hCMV_-driven transposase SB100X expression vector (P_hCMV_-SB100X-pA) | Addgene (no. 13367) |
| pXS140 | Lentiviral vector encoding constitutive P_EF1a_-driven mammalian  expression units for mouse Sox2 (LTR-P_hCMV_-mSox2-pA-LTR). | This study |
| pXS141 | Lentiviral vector encoding constitutive P_EF1a_-driven mammalian  expression units for mouse Oct4 (LTR-P_hCMV_-mOct4-pA-LTR). | This study |
| pZW53 | Red light induced NanoLuc expression vector (P_RL_-NanoLuc-pA; P_RL,_ 5×UAS-P_hCMVmin_-kozak) | This study |
| pQL259 | Red light induced NanoLuc and IFN-β expression vector (P_RL_- NanoLuc-P2A-IFN-β-pA; P_RL,_ 5×UAS-P_TATA_-kozak) | This study |
| pQL282 | Red light induced NanoLuc and IFN-β expression vector (P_RL_-NanoLuc-P2A-IFN-β-pA; P_RL,_ 5×UAS-P_hCMVmin_) | This study |
| pNX175 | Sleeping Beauty transposase expression vector encoding constitutive 2NLS-LDB3-p65-HSF1 fusion protein utilizing a PuroR resistance cassette (ITR-P_EF1α_-2NLS-LDB3-p65-HSF1-pA::P_mPGK_-PuroR-pA-ITR) | This study |
| pNX176 | Sleeping Beauty transposase expression vector encoding constitutive Gal4 DBD-FnBphP fusion protein, RL induced IFN-β and NanoLuc utilizing a ZeoR resistance cassette (ITR-P_EF1α_-Gal4 DBD-FnBphP-pA::P_RL_-IFN-β-P2A-NanoLuc-pA::P_mPGK_-ZeoR-P2A-EGFP-pA-ITR; P_RL_, 5×UAS-P_hCMVmin_-kozak) | This study |
| pNX12 | Constitutive 2NLS-LDB3-p65-HSF1 fusion protein expression vector (P_hCMV_-2NLS-LDB3-p65-HSF1-pA). | This study |
| pNX13 | Constitutive 1NLS-LDB3-p65-HSF1 fusion protein expression vector (P_hCMV_-1NLS-LDB3-p65-HSF1-pA). | This study |
| pQL256 | Constitutive LDB3-p65-HSF1 fusion protein expression vector (P_hCMV_-LDB3-p65-HSF1-pA) | This study |
| pQL217 | Constitutive Gal4 DBD-*Dr*BphP fusion protein expression vector (P_hCMV_-Gal4 DBD-*Dr*BphP-pA) | This study |
| pQL232 | Constitutive 3NLS-LDB3-VP64 fusion protein expression vector (P_hCMV_-3NLS-LDB3-VP64-pA). | This study |
| pQL233 | Constitutive 3NLS-LDB3-p65 fusion protein expression vector (P_hCMV_-3NLS-LDB3-p65-pA). | This study |
| pQL234 | Constitutive 3NLS-LDB3-VP16 fusion protein expression vector (P_hCMV_-3NLS-LDB3-VP16-pA). | This study |
| pQL235 | Constitutive 3NLS-LDB3-VPR fusion protein expression vector (P_hCMV_-3NLS-LDB3-VPR-pA). | This study |
| pQL236 | Constitutive 3NLS-LDB3-p65-HSF1 fusion protein expression vector (P_hCMV_-3NLS-LDB3-p65-HSF1-pA). | This study |
| pQL256 | Constitutive LDB3-p65-HSF1 fusion protein expression vector (P_hCMV_-LDB3-p65-HSF1-pA). | This study |
| pQL325 | Constitutive Gal4 DBD-PnBphP fusion protein expression vector (P_hCMV_-Gal4 DBD-PnBphP-pA). | This study |
| pQL326 | Constitutive Gal4 DBD-FnBphP fusion protein expression vector (P_hCMV_-Gal4 DBD-FnBphP-pA). | This study |
| pNX228 | Sleeping Beauty transposase expression vector encoding constitutive IFN-β fusion protein utilizing a ZeoR resistance cassette (ITR-P_EF1α_-IFN-β-pA::P_mPGK_-ZeoR-P2A-EGFP-pA-ITR) | This study |

**Abbreviations**: **APCs**, antigen-presenting cells; **ALT**, alanine aminotransferase; **AST**, aspartate aminotransferase; **BUN**, urea nitrogen; **BV**, biliverdin; **CpG,** cytidine phosphate guanosine; **CRE**, creatinine; **DCs**, dendritic cells; ***Dr*BphP**, *Deinococcus radiodurans* bacteriophytochrome; **EGFP**, enhanced green fluorescent protein; **FnBphP**, chimeric photosensory protein fusing the N-terminal extension (NTE) of FphA to N-terminus of  *Dr*BphP-PCM; **FRL**, far-red light; **Gal4 DBD**, an N-terminal yeast Gal4 DNA binding domain; **H&E**, hematoxylin and eosin; **HGB**, hemoglobin; **HCT**, hematocrit; **iPSC**, induced pluripotent stem cell; **ICD**, immunogenic cell death; **IL-6,** interlukine-6; **IFN-γ**, type I interferon γ; **IFN-β**, type I interferon β; **MCS**, multiple cloning site; **MEFs**, mouse embryonic fibroblasts; **NIR**, near-infrared; **NanoLuc**, nanoluciferase; **NLS**, nuclear localization signal; **NK**, natural killer; **NTE**, N-terminal extension; **Oct4**, octamer-binding transcription factor 4; **P_hCMV_**, human cytomegalovirus immediate early promoter; **P_EF1α_**, human elongation factor 1α promoter ; **P_mPGK_**, mouse phosphoglycerate kinase 1 promoter; **P_hCMVmin_**, minimal version of P_hCMV_; **PCM**, photosensory core module ; **p65-HSF1**, the 65-kDa transactivator subunit of NF-κB (p65) and heat shock factor 1 (HSF1) transactivation domains; **PnBphP**, chimeric photosensory protein fusing the N-terminal extension (NTE) of PhyA to N-terminus of  *Dr*BphP-PCM; **PuroR**, gene product that confers puromycin resistance to mammalian cells; **PhyA**, phytochrome A; **PBS**, phosphate-buffered saline; **PLT**, platelets; **PCT**, platelet crit; **RID**, red light-induced protein dimerization; **RIVA**, a red/far-red light controlled iPSC-based vaccines; **RL**, red light; **RBC**, red blood cells; **Sox2**, SRY-box containing gene 2; **SB100X**, Sleeping Beauty transposase; **TAAs**, tumor associated antigens; **TLR7/8**, toll-like receptor 7/8; **TLR9,** toll-like receptor 9; **TNF-α**, tumor necrosis factor-α; **TSAs**, tumor-specific antigens;, **UAS**, Gal4 DBD-specific binding sequence; **VP16**, a *Herpes simplex* virus (HSV)-derived transcriptional activator protein; **VP64**, a tetrameric repeat of the minimal activation domain derived from the *Herpes simplex* virus protein VP16; **VPR**, a tripartite activator VP64-p65-Rta fusion protein; **VPA**, valproic acid; **WBC**, white blood cells; **ZeoR**, gene product that confers zeocin resistance to mammalian cells.

**Table S2.** **Expression vectors and transfection mixtures used in this study**

| plasmid  (ng) | Fig.2B  VP16 | Fig.2B  VP64 | Fig.2B  p65 | Fig.2B  VPR | Fig.2B  p65-HSF1 |
| --- | --- | --- | --- | --- | --- |
| pQL259 | 200 | 200 | 200 | 200 | 200 |
| pQL217 | 150 | 150 | 150 | 150 | 150 |
| pQL234 | 200 | 0 | 0 | 0 | 0 |
| pQL232 | 0 | 200 | 0 | 0 | 0 |
| pQL233 | 0 | 0 | 200 | 0 | 0 |
| pQL235 | 0 | 0 | 0 | 200 | 0 |
| pQL236 | 0 | 0 | 0 | 0 | 200 |
| Total mount | 550 | 550 | 550 | 550 | 550 |

| plasmid  (ng) | Fig.2C  0NLS-LDB3- p65-HSF1 | Fig.2C  1NLS-LDB3- p65-HSF1 | Fig.2C  2NLS-LDB3- p65-HSF1 | Fig.2C  3NLS-LDB3- p65-HSF1 |
| --- | --- | --- | --- | --- |
| pQL259 | 200 | 200 | 200 | 200 |
| pQL217 | 150 | 150 | 150 | 150 |
| pQL256 | 200 | 0 | 0 | 0 |
| pNX13 | 0 | 200 | 0 | 0 |
| pNX12 | 0 | 0 | 200 | 0 |
| pQL236 | 0 | 0 | 0 | 200 |
| Total mount | 550 | 550 | 550 | 550 |

| plasmid  (ng) | Fig.2D  P_RL1_ | Fig. 2D  P_RL2_ | Fig. 2D  P_RL3_ |
| --- | --- | --- | --- |
| pNX12 | 200 | 200 | 200 |
| pQL217 | 150 | 150 | 150 |
| pQL259 | 200 | 0 | 0 |
| pQL282 | 0 | 200 | 0 |
| pZW53 | 0 | 0 | 200 |
| Total mount | 550 | 550 | 550 |

| plasmid  (ng) | Fig.2E  DrBphP | Fig. 2E  PnBphP | Fig. 2E  FnBphP |
| --- | --- | --- | --- |
| pNX12 | 200 | 200 | 200 |
| pZW53 | 200 | 200 | 200 |
| pQL217 | 150 | 0 | 0 |
| pQL325 | 0 | 150 | 0 |
| pQL326 | 0 | 0 | 150 |
| Total mount | 550 | 550 | 550 |

| plasmid  (ng) | Fig.S2  FnBphP |
| --- | --- |
| pNX12 | 200 |
| pZW53 | 200 |
| pQL326 | 150 |
| Total mount | 550 |

**Table S3.** DNA sequence information of RID and RIVA

| **pNX12: P_hCMV_-2NLS-LDB3-p65-HSF1-bGH polyA** |
| --- |
| **gttgacattgattattgactagttattaatagtaatcaattacggggtcattagttcatagcccatatatggagttccgcgttacataacttacggtaaatggcccgcctggctgaccgcccaacgacccccgcccattgacgtcaataatgacgtatgttcccatagtaacgccaatagggactttccattgacgtcaatgggtggagtatttacggtaaactgcccacttggcagtacatcaagtgtatcatatgccaagtacgccccctattgacgtcaatgacggtaaatggcccgcctggcattatgcccagtacatgaccttatgggactttcctacttggcagtacatctacgtattagtcatcgctattaccatggtgatgcggttttggcagtacatcaatgggcgtggatagcggtttgactcacggggatttccaagtctccaccccattgacgtcaatgggagtttgttttggcaccaaaatcaacgggactttccaaaatgtcgtaacaactccgccccattgacgcaaatgggcggtaggcgtgtacggtgggaggtctatataagcagagctctctggctaactagagaacccactgcttactggcttatcgaaattaatacgactcactatagggagacccaagctggctagcgtttaaacttaagcttggtaccgccaccatgggatccccgaagaaaaagcggaaagtcgaggcctccgcatctccaaaaaaaaaaagcaaggttgaagcatctggatccggtaccggaggaagtggcagctctggcggcagtggagggtctggtggcagcggaATGGAAGTTCAGCTGCAGGCAAGCGGTGGTGGTTTTGTTCAGCCTGGTGGTAGCCTGCGTCTGAGCTGTGCAGCCAGCGGTTTTACCTGGGATCATTACATCATGGGCTGGTTTCGCCAGGCACCGGGTAAAGAACGTGAATTTGTTAGCGCAATCAGCGAAAATGGTGATGCATGGAATTATTATGCCGATAGCGTGAAAGGTCGCTTTACCATTAGCCGTGATAATAGCAAAAATACCGTTTACCTGCAGATGAATAGTCTGCGTGCAGAAGATACCGCAACCTATTATTGTGCAATCGGTTTTGATGTTCCATCTGGTCGTTCTTGGCAGGGTTCTCATTTTTGGATGTATTGGGGTCAGGGCACCCAGGTTACCGTTAGCAGCTCGGACAGCGCCGGCAGCGCCGGCAGCGCCGGCAGCGGATCCCCTTCAGGGCAGATCAGCAACCAGGCCCTGGCTCTGGCCCCTAGCTCCGCTCCAGTGCTGGCCCAGACTATGGTGCCCTCTAGTGCTATGGTGCCTCTGGCCCAGCCACCTGCTCCAGCCCCTGTGCTGACCCCAGGACCACCCCAGTCACTGAGCGCTCCAGTGCCCAAGTCTACACAGGCCGGCGAGGGGACTCTGAGTGAAGCTCTGCTGCACCTGCAGTTCGACGCTGATGAGGACCTGGGAGCTCTGCTGGGGAACAGCACCGATCCCGGAGTGTTCACAGATCTGGCCTCCGTGGACAACTCTGAGTTTCAGCAGCTGCTGAATCAGGGCGTGTCCATGTCTCATAGTACAGCCGAACCAATGCTGATGGAGTACCCCGAAGCCATTACCCGGCTGGTGACCGGCAGCCAGCGGCCCCCCGACCCCGCTCCAACTCCCCTGGGAACCAGCGGCCTGCCTAATGGGCTGTCCGGAGATGAAGACTTCTCAAGCATCGCTGATATGGACTTTAGTGCCCTGCTGTCACAGATTTCCTCTAGTGGGCAGGGAGGAGGTGGAAGCGGCTTCAGCGTGGACACCAGTGCCCTGCTGGACCTGTTCAGCCCCTCGGTGACCGTGCCCGACATGAGCCTGCCTGACCTTGACAGCAGCCTGGCCAGTATCCAAGAGCTCCTGTCTCCCCAGGAGCCCCCCAGGCCTCCCGAGGCAGAGAACAGCAGCCCGGATTCAGGGAAGCAGCTGGTGCACTACACAGCGCAGCCGCTGTTCCTGCTGGACCCCGGCTCCGTGGACACCGGGAGCAACGACCTGCCGGTGCTGTTTGAGCTGGGAGAGGGCTCCTACTTCTCCGAAGGGGACGGCTTCGCCGAGGACCCCACCATCTCCCTGCTGACAGGCTCGGAGCCTCCCAAAGCCAAGGACCCCACTGTCTCCTAATctagagggcccgtttaaacccgctgatcagcctcgactgtgccttctagttgccagccatctgttgtttgcccctcccccgtgccttccttgaccctggaaggtgccactcccactgtcctttcctaataaaatgaggaaattgcatcgcattgtctgagtaggtgtcattctattctggggggtggggtggggcaggacagcaagggggaggattgggaagacaatagcaggcatgctggggatgcggtgggctctatgg** |
| **pQL217: P_hCMV_-Gal4 DBD-DrBphP-bGH polyA** |
| **gttgacattgattattgactagttattaatagtaatcaattacggggtcattagttcatagcccatatatggagttccgcgttacataacttacggtaaatggcccgcctggctgaccgcccaacgacccccgcccattgacgtcaataatgacgtatgttcccatagtaacgccaatagggactttccattgacgtcaatgggtggagtatttacggtaaactgcccacttggcagtacatcaagtgtatcatatgccaagtacgccccctattgacgtcaatgacggtaaatggcccgcctggcattatgcccagtacatgaccttatgggactttcctacttggcagtacatctacgtattagtcatcgctattaccatggtgatgcggttttggcagtacatcaatgggcgtggatagcggtttgactcacggggatttccaagtctccaccccattgacgtcaatgggagtttgttttggcaccaaaatcaacgggactttccaaaatgtcgtaacaactccgccccattgacgcaaatgggcggtaggcgtgtacggtgggaggtctatataagcagagctctctggctaactagagaacccactgcttactggcttatcgaaattaatacgactcactatagggagacccaagctggctagcgtttaaacttaagcttggtaccGCCACCATGTGCGGCCGCAAGCTGCTGAGCAGCATCGAGCAGGCCTGCGACATCTGCAGGCTGAAGAAGCTGAAGTGTAGCAAGGAGAAGCCTAAGTGCGCCAAGTGCCTGAAGAACAACTGGGAGTGCAGGTATAGCCCCAAGACCAAGAGGAGCCCCCTGACCAGAGCCCACCTGACCGAAGTGGAGAGCAGGCTGGAGAGGCTGGAGCAGCTGTTCCTGCTGATCTTCCCCAGAGAAGACCTGGACATGATCCTGAAGATGGACAGCCTGCAGGATATCAAGGCCCTCCTGACAGGCCTGTTTGTCCAGGACAACGTCAACAAGGACGCCGTGACCGATAGACTGGCCTCTGTGGAGACAGACATGCCCCTGACCCTGAGGCAGCACAGGATCAGCGCCACCAGCTCCAGCGAGGAGTCCAGCAACAAGGGCCAGAGGCAGCTCACCGTGAGCGCCAGCGGAAGTGGCGGAGGAGGCGACGTCATGAGCAGAGATCCCCTGCCTTTCTTCCCACCACTGTACCTCGGAGGCCCTGAGATCACCACCGAGAACTGCGAGAGAGAGCCCATTCACATCCCCGGCTCTATTCAGCCTCATGGCGCTCTGCTGACAGCCGATGGACATTCTGGCGAGGTGCTGCAGATGAGCCTGAATGCCGCCACCTTCCTGGGCCAAGAACCTACCGTTCTGAGAGGCCAGACACTGGCTGCACTGCTGCCTGAACAATGGCCTGCTCTGCAAGCTGCTCTGCCTCCTGGATGTCCTGACGCTCTGCAGTACAGAGCCACACTGGATTGGCCTGCCGCCGGACATCTGTCTCTGACAGTGCACAGAGTGGGCGAGCTGCTGATCCTGGAATTCGAGCCTACAGAGGCCTGGGACTCTACAGGACCTCACGCTCTGAGAAACGCCATGTTCGCCCTGGAAAGCGCCCCTAATCTGAGAGCCCTGGCCGAAGTGGCTACCCAGACAGTCAGAGAGCTGACCGGCTTCGACAGAGTGATGCTGTACAAGTTCGCCCCTGACGCCACCGGCGAAGTGATTGCCGAAGCCAGAAGAGAAGGCCTGCACGCCTTTCTGGGCCACAGATTTCCAGCCAGCGACATCCCTGCTCAGGCTAGAGCCCTGTACACCCGGCATCTGCTGAGACTGACCGCCGATACAAGAGCCGCTGCTGTGCCACTGGACCCCGTTCTGAACCCTCAGACAAACGCCCCTACACCTCTTGGCGGAGCTGTGCTGAGAGCCACCTCTCCTATGCACATGCAGTACCTGCGGAACATGGGCGTGGGAAGCAGCCTGTCTGTGTCTGTGGTTGTTGGCGGACAGCTGTGGGGACTGATCGCCTGTCATCACCAGACACCTTACGTGCTGCCTCCAGACCTGCGGACAACCCTGGAATATCTGGGCAGACTGCTGAGCCTGCAGGTCCAAGTGAAAGAGGCTGCTGACGTCGCCGCCTTCAGACAGAGCCTGAGAGAACACCATGCCAGAGTGGCACTGGCCGCTGCTCATTCTCTGAGCCCTCACGATACCCTGAGCGACCCTGCTCTGGATCTGCTGGGACTTATGAGAGCCGGCGGACTGATCCTGAGATTTGAAGGCAGATGGCAGACCCTGGGAGAAGTGCCTCCTGCTCCTGCTGTTGATGCTCTGCTGGCCTGGCTCGAAACACAACCTGGTGCTCTGGTGCAGACAGATGCCCTGGGACAACTTTGGCCTGCTGGCGCTGATCTGGCTCCTTCTGCTGCTGGACTGCTCGCCATCTCTGTTGGAGAAGGCTGGAGCGAGTGTCTCGTGTGGCTCAGACCTGAGCTGAGGCTGGAAGTTGCTTGGGGCGGAGCTACACCCGATCAGGCCAAGGATGATCTGGGCCCCAGACACAGCTTCGACACCTACCTGGAAGAGAAGCGGGGCTATGCCGAACCTTGGCACCCTGGCGAAATTGAGGAAGCCCAGGACCTGAGGGACACACTGACAGGTGCTCTTTAAtctagagggcccgtttaaacccgctgatcagcctcgactgtgccttctagttgccagccatctgttgtttgcccctcccccgtgccttccttgaccctggaaggtgccactcccactgtcctttcctaataaaatgaggaaattgcatcgcattgtctgagtaggtgtcattctattctggggggtggggtggggcaggacagcaagggggaggattgggaagacaatagcaggcatgctggggatgcggtgggctctatgg** |
| **pQL325: P_hCMV_-Gal4 DBD-PnBphP-bGH polyA** |
| **gttgacattgattattgactagttattaatagtaatcaattacggggtcattagttcatagcccatatatggagttccgcgttacataacttacggtaaatggcccgcctggctgaccgcccaacgacccccgcccattgacgtcaataatgacgtatgttcccatagtaacgccaatagggactttccattgacgtcaatgggtggagtatttacggtaaactgcccacttggcagtacatcaagtgtatcatatgccaagtacgccccctattgacgtcaatgacggtaaatggcccgcctggcattatgcccagtacatgaccttatgggactttcctacttggcagtacatctacgtattagtcatcgctattaccatggtgatgcggttttggcagtacatcaatgggcgtggatagcggtttgactcacggggatttccaagtctccaccccattgacgtcaatgggagtttgttttggcaccaaaatcaacgggactttccaaaatgtcgtaacaactccgccccattgacgcaaatgggcggtaggcgtgtacggtgggaggtctatataagcagagctctctggctaactagagaacccactgcttactggcttatcgaaattaatacgactcactatagggagacccaagctggctagcgtttaaacttaagcttggtaccGCCACCATGTGCGGCCGCAAGCTGCTGAGCAGCATCGAGCAGGCCTGCGACATCTGCAGGCTGAAGAAGCTGAAGTGTAGCAAGGAGAAGCCTAAGTGCGCCAAGTGCCTGAAGAACAACTGGGAGTGCAGGTATAGCCCCAAGACCAAGAGGAGCCCCCTGACCAGAGCCCACCTGACCGAAGTGGAGAGCAGGCTGGAGAGGCTGGAGCAGCTGTTCCTGCTGATCTTCCCCAGAGAAGACCTGGACATGATCCTGAAGATGGACAGCCTGCAGGATATCAAGGCCCTCCTGACAGGCCTGTTTGTCCAGGACAACGTCAACAAGGACGCCGTGACCGATAGACTGGCCTCTGTGGAGACAGACATGCCCCTGACCCTGAGGCAGCACAGGATCAGCGCCACCAGCTCCAGCGAGGAGTCCAGCAACAAGGGCCAGAGGCAGCTCACCGTGAGCGCCAGCGGAAGTGGCGGAGGAGGCGACGTCATGGAGAAGAAGATGAGCGGATCTCGTCCCACACAGTCCAGCGAGGGATCTCGTAGATCTCGTCACTCCGCTCGTATCATCGCTCAGACCACCGTGGACGCCAAACTGCACGCCGATTTCGAGGAGAGCGGCTCCTCCTTTGATTACTCCACCAGCGTGAGGGTGACTGGTCCCGTGGTCGAGAACCAGCCTCCTAGGAGCGACAAGGTCACCACAACCTACCTCCATCATATCCAGAAGGGCAAGCTGATCCAGCCCTTTGGATGTTTACTGGCTTTAGACGAAAAGACCTTCAAGGTCATGAGCAGAGATCCCCTGCCTTTCTTCCCACCACTGTACCTCGGAGGCCCTGAGATCACCACCGAGAACTGCGAGAGAGAGCCCATTCACATCCCCGGCTCTATTCAGCCTCATGGCGCTCTGCTGACAGCCGATGGACATTCTGGCGAGGTGCTGCAGATGAGCCTGAATGCCGCCACCTTCCTGGGCCAAGAACCTACCGTTCTGAGAGGCCAGACACTGGCTGCACTGCTGCCTGAACAATGGCCTGCTCTGCAAGCTGCTCTGCCTCCTGGATGTCCTGACGCTCTGCAGTACAGAGCCACACTGGATTGGCCTGCCGCCGGACATCTGTCTCTGACAGTGCACAGAGTGGGCGAGCTGCTGATCCTGGAATTCGAGCCTACAGAGGCCTGGGACTCTACAGGACCTCACGCTCTGAGAAACGCCATGTTCGCCCTGGAAAGCGCCCCTAATCTGAGAGCCCTGGCCGAAGTGGCTACCCAGACAGTCAGAGAGCTGACCGGCTTCGACAGAGTGATGCTGTACAAGTTCGCCCCTGACGCCACCGGCGAAGTGATTGCCGAAGCCAGAAGAGAAGGCCTGCACGCCTTTCTGGGCCACAGATTTCCAGCCAGCGACATCCCTGCTCAGGCTAGAGCCCTGTACACCCGGCATCTGCTGAGACTGACCGCCGATACAAGAGCCGCTGCTGTGCCACTGGACCCCGTTCTGAACCCTCAGACAAACGCCCCTACACCTCTTGGCGGAGCTGTGCTGAGAGCCACCTCTCCTATGCACATGCAGTACCTGCGGAACATGGGCGTGGGAAGCAGCCTGTCTGTGTCTGTGGTTGTTGGCGGACAGCTGTGGGGACTGATCGCCTGTCATCACCAGACACCTTACGTGCTGCCTCCAGACCTGCGGACAACCCTGGAATATCTGGGCAGACTGCTGAGCCTGCAGGTCCAAGTGAAAGAGGCTGCTGACGTCGCCGCCTTCAGACAGAGCCTGAGAGAACACCATGCCAGAGTGGCACTGGCCGCTGCTCATTCTCTGAGCCCTCACGATACCCTGAGCGACCCTGCTCTGGATCTGCTGGGACTTATGAGAGCCGGCGGACTGATCCTGAGATTTGAAGGCAGATGGCAGACCCTGGGAGAAGTGCCTCCTGCTCCTGCTGTTGATGCTCTGCTGGCCTGGCTCGAAACACAACCTGGTGCTCTGGTGCAGACAGATGCCCTGGGACAACTTTGGCCTGCTGGCGCTGATCTGGCTCCTTCTGCTGCTGGACTGCTCGCCATCTCTGTTGGAGAAGGCTGGAGCGAGTGTCTCGTGTGGCTCAGACCTGAGCTGAGGCTGGAAGTTGCTTGGGGCGGAGCTACACCCGATCAGGCCAAGGATGATCTGGGCCCCAGACACAGCTTCGACACCTACCTGGAAGAGAAGCGGGGCTATGCCGAACCTTGGCACCCTGGCGAAATTGAGGAAGCCCAGGACCTGAGGGACACACTGACAGGTGCTCTTTAAtctagagggcccgtttaaacccgctgatcagcctcgactgtgccttctagttgccagccatctgttgtttgcccctcccccgtgccttccttgaccctggaaggtgccactcccactgtcctttcctaataaaatgaggaaattgcatcgcattgtctgagtaggtgtcattctattctggggggtggggtggggcaggacagcaagggggaggattgggaagacaatagcaggcatgctggggatgcggtgggctctatgg** |
| **pQL326: P_hCMV_-Gal4 DBD-FnBphP-bGH polyA** |
| **gttgacattgattattgactagttattaatagtaatcaattacggggtcattagttcatagcccatatatggagttccgcgttacataacttacggtaaatggcccgcctggctgaccgcccaacgacccccgcccattgacgtcaataatgacgtatgttcccatagtaacgccaatagggactttccattgacgtcaatgggtggagtatttacggtaaactgcccacttggcagtacatcaagtgtatcatatgccaagtacgccccctattgacgtcaatgacggtaaatggcccgcctggcattatgcccagtacatgaccttatgggactttcctacttggcagtacatctacgtattagtcatcgctattaccatggtgatgcggttttggcagtacatcaatgggcgtggatagcggtttgactcacggggatttccaagtctccaccccattgacgtcaatgggagtttgttttggcaccaaaatcaacgggactttccaaaatgtcgtaacaactccgccccattgacgcaaatgggcggtaggcgtgtacggtgggaggtctatataagcagagctctctggctaactagagaacccactgcttactggcttatcgaaattaatacgactcactatagggagacccaagctggctagcgtttaaacttaagcttggtaccGCCACCATGTGCGGCCGCAAGCTGCTGAGCAGCATCGAGCAGGCCTGCGACATCTGCAGGCTGAAGAAGCTGAAGTGTAGCAAGGAGAAGCCTAAGTGCGCCAAGTGCCTGAAGAACAACTGGGAGTGCAGGTATAGCCCCAAGACCAAGAGGAGCCCCCTGACCAGAGCCCACCTGACCGAAGTGGAGAGCAGGCTGGAGAGGCTGGAGCAGCTGTTCCTGCTGATCTTCCCCAGAGAAGACCTGGACATGATCCTGAAGATGGACAGCCTGCAGGATATCAAGGCCCTCCTGACAGGCCTGTTTGTCCAGGACAACGTCAACAAGGACGCCGTGACCGATAGACTGGCCTCTGTGGAGACAGACATGCCCCTGACCCTGAGGCAGCACAGGATCAGCGCCACCAGCTCCAGCGAGGAGTCCAGCAACAAGGGCCAGAGGCAGCTCACCGTGAGCGCCAGCGGAAGTGGCGGAGGAGGCGACGTCATGAGCGAGCTGCCCAGCAGATCCATCTCCCCTAGGGACCCTAGCCCCGGCGAGACCCCAGGAAGAGACCCTTCCACCCCTAGCACCGACGCCGGCGTGGGATACAGCGCCAGCCAGGATGCCCCCTCCTTTGGCGCCTACGATAGGGTGTACCCCATCAGGAGCCTGGTGAGCCTGGAGCCTCCCGCCACATCCGAGCCCTCCAGCAACAAGTCCAAGTCCCCTCTGAGCCCTACATCCGGCGCCAGACAGTTTTCCATCATCGACGGCCACACATGGACCAGGCTGAGAAGCGATTCCAGGGCCAACTCCACCGACTACTCCGGCGGCACAGGCCTGTCCCCCGAGTCTAGCGAGGCCCCTTCCAGCCAGAGGATGTCCGACTCCAGCTCCGCCAGGCCTCCCTCCAACACCACAGGCCTGAGGAGAGGCGATGATCACACCACCTTTACCCCTAGCAGCGAGGACAGCCACCCTCAGGTGCAGGAGCCCTACGAGCTGATGACCACAAGGTTTAGACACGTGGTGACCGATGACGGCCACGCCGTGATCACCGGCAGGACCGTGGATAGCTTTAAGGCcATGAGCAGAGATCCCCTGCCTTTCTTCCCACCACTGTACCTCGGAGGCCCTGAGATCACCACCGAGAACTGCGAGAGAGAGCCCATTCACATCCCCGGCTCTATTCAGCCTCATGGCGCTCTGCTGACAGCCGATGGACATTCTGGCGAGGTGCTGCAGATGAGCCTGAATGCCGCCACCTTCCTGGGCCAAGAACCTACCGTTCTGAGAGGCCAGACACTGGCTGCACTGCTGCCTGAACAATGGCCTGCTCTGCAAGCTGCTCTGCCTCCTGGATGTCCTGACGCTCTGCAGTACAGAGCCACACTGGATTGGCCTGCCGCCGGACATCTGTCTCTGACAGTGCACAGAGTGGGCGAGCTGCTGATCCTGGAATTCGAGCCTACAGAGGCCTGGGACTCTACAGGACCTCACGCTCTGAGAAACGCCATGTTCGCCCTGGAAAGCGCCCCTAATCTGAGAGCCCTGGCCGAAGTGGCTACCCAGACAGTCAGAGAGCTGACCGGCTTCGACAGAGTGATGCTGTACAAGTTCGCCCCTGACGCCACCGGCGAAGTGATTGCCGAAGCCAGAAGAGAAGGCCTGCACGCCTTTCTGGGCCACAGATTTCCAGCCAGCGACATCCCTGCTCAGGCTAGAGCCCTGTACACCCGGCATCTGCTGAGACTGACCGCCGATACAAGAGCCGCTGCTGTGCCACTGGACCCCGTTCTGAACCCTCAGACAAACGCCCCTACACCTCTTGGCGGAGCTGTGCTGAGAGCCACCTCTCCTATGCACATGCAGTACCTGCGGAACATGGGCGTGGGAAGCAGCCTGTCTGTGTCTGTGGTTGTTGGCGGACAGCTGTGGGGACTGATCGCCTGTCATCACCAGACACCTTACGTGCTGCCTCCAGACCTGCGGACAACCCTGGAATATCTGGGCAGACTGCTGAGCCTGCAGGTCCAAGTGAAAGAGGCTGCTGACGTCGCCGCCTTCAGACAGAGCCTGAGAGAACACCATGCCAGAGTGGCACTGGCCGCTGCTCATTCTCTGAGCCCTCACGATACCCTGAGCGACCCTGCTCTGGATCTGCTGGGACTTATGAGAGCCGGCGGACTGATCCTGAGATTTGAAGGCAGATGGCAGACCCTGGGAGAAGTGCCTCCTGCTCCTGCTGTTGATGCTCTGCTGGCCTGGCTCGAAACACAACCTGGTGCTCTGGTGCAGACAGATGCCCTGGGACAACTTTGGCCTGCTGGCGCTGATCTGGCTCCTTCTGCTGCTGGACTGCTCGCCATCTCTGTTGGAGAAGGCTGGAGCGAGTGTCTCGTGTGGCTCAGACCTGAGCTGAGGCTGGAAGTTGCTTGGGGCGGAGCTACACCCGATCAGGCCAAGGATGATCTGGGCCCCAGACACAGCTTCGACACCTACCTGGAAGAGAAGCGGGGCTATGCCGAACCTTGGCACCCTGGCGAAATTGAGGAAGCCCAGGACCTGAGGGACACACTGACAGGTGCTCTTTAAtctagagggcccgtttaaacccgctgatcagcctcgactgtgccttctagttgccagccatctgttgtttgcccctcccccgtgccttccttgaccctggaaggtgccactcccactgtcctttcctaataaaatgaggaaattgcatcgcattgtctgagtaggtgtcattctattctggggggtggggtggggcaggacagcaagggggaggattgggaagacaatagcaggcatgctggggatgcggtgggctctatgg** |
| **pZW53: P_RL_-NanoLuc-bGH polyA; P_RL,_ 5×UAS-P_hCMVmin_-kozak** |
| **CGGAGTACTGTCCTCCGAGCGGAGTACTGTCCTCCGAGCGGAGTACTGTCCTCCGAGCGGAGTACTGTCCTCCGAGCGGAGTACTGTCCTCCGAGCctgcaggTCGAGCTCGGTACCCGGGTCGAGTAGGCGTGTACGGTGGGAGGCCTATATAAGCAGAGCTCGTTTAGTGAACCGTCAGATCGCCTGGAGACGCCATCCACGCTGTTTTGACCTCCATAGAAGACACCGGGACCGATCCAGCCTCCGCGgAATTCGAGCTCGCCCGGGGATCCGCCACCATGACTAGTGAGACAGACACACTCCTGCTATGGGTACTGCTGCTCTGGGTTCCAGGTTCCACTGGTGACGCTAGTggtggttctggtATGGTCTTCACACTCGAAGATTTCGTTGGGGACTGGCGACAGACAGCCGGCTACAACCTGGACCAAGTCCTTGAACAGGGAGGTGTGTCCAGTTTGTTTCAGAATCTCGGGGTGTCCGTAACTCCGATCCAAAGGATTGTCCTGAGCGGTGAAAATGGGCTGAAGATCGACATCCATGTCATCATCCCGTATGAAGGTCTGAGCGGCGACCAAATGGGCCAGATCGAAAAAATTTTTAAGGTGGTGTACCCTGTGGATGATCATCACTTTAAGGTGATCCTGCACTATGGCACACTGGTAATCGACGGGGTTACGCCGAACATGATCGACTATTTCGGACGGCCGTATGAAGGCATCGCCGTGTTCGACGGCAAAAAGATCACTGTAACAGGGACCCTGTGGAACGGCAACAAAATTATCGACGAGCGCCTGATCAACCCCGACGGCTCCCTGCTGTTCCGAGTAACCATCAACGGAGTGACCGGCTGGCGGCTGTGCGAACGCATTCTGGCGgCTAGTTCCGGTTGTAAGCCTTGCATATGTACAGTCCCAGAAGTATCATCTGTCTTCATCTTCCCCCCAAAGCCCAAGGATGTGCTCACCATTACTCTGACTCCTAAGGTCACGTGTGTTGTGGTAGACATCAGCAAGGATGATCCCGAGGTCCAGTTCAGCTGGTTTGTAGATGATGTGGAGGTGCACACAGCTCAGACGCAACCCCGGGAGGAGCAGTTCAACAGCACTTTCCGCTCAGTCAGTGAACTTCCCATCATGCACCAGGACTGGCTCAATGGCAAGGAGTTCAAATGCAGGGTCAACAGTGCAGCTTTCCCTGCCCCCATCGAGAAAACCATCTCCAAAACCAAAGGCAGACCGAAGGCTCCACAGGTGTACACCATTCCACCTCCCAAGGAGCAGATGGCCAAGGATAAAGTCAGTCTGACCTGCATGATAACAGACTTCTTCCCTGAAGACATTACTGTGGAGTGGCAGTGGAATGGGCAGCCAGCGGAGAACTACAAGAACACTCAGCCCATCATGGACACAGATGGCTCTTACTTCGTCTACAGCAAGCTCAATGTGCAGAAGAGCAACTGGGAGGCAGGAAATACTTTCACCTGCTCTGTGTTACATGAGGGCCTGCACAACCACCATACTGAGAAGAGCCTCTCCCACTCTCCTGGTAAAgCTAGCGGATCCACCGGTgTCTAGTAATCTAGAcagacatgataagatacattgatgagtttggacaaaccacaactagaatgcagtgaaaaaaatgctttatttgtgaaatttgtgatgctattgctttatttgtaaccattataagctgcaataaacaagttaacaacaacaattgcattcattttatgtttcaggttcagggggaggtgtgggaggtttttt** |
| **pNX175: ITR-P_EF1α_-2NLS-LDB3-p65-HSF1-SV40 polyA :: P_mPGK_-PuroR-bGH polyA-ITR** |
| **gggaaagtgatgtcgtgtactggctccgcctttttcccgagggtgggggagaaccgtatataagtgcagtagtcgccgtgaacgttctttttcgcaacgggtttgccgccagaacacaggtaagtgccgtgtgtggttcccgcgggcctggcctctttacgggttatggcccttgcgtgccttgaattacttccacctggctgcagtacgtgattcttgatcccgagcttcgggttggaagtgggtgggagagttcgaggccttgcgcttaaggagccccttcgcctcgtgcttgagttgaggcctggcctgggcgctggggccgccgcgtgcgaatctggtggcaccttcgcgcctgtctcgctgctttcgataagtctctagccatttaaaatttttgatgacctgctgcgacgctttttttctggcaagatagtcttgtaaatgcgggccaagatctgcacactggtatttcggtttttggggccgcgggcggcgacggggcccgtgcgtcccagcgcacatgttcggcgaggcggggcctgcgagcgcggccaccgagaatcggacgggggtagtctcaagctggccggcctgctctggtgcctggcctcgcgccgccgtgtatcgccccgccctgggcggcaaggctggcccggtcggcaccagttgcgtgagcggaaagatggccgcttcccggccctgctgcagggagctcaaaatggaggacgcggcgctcgggagagcgggcgggtgagtcacccacacaaaggaaaagggcctttccgtcctcagccgtcgcttcatgtgactccacggagtaccgggcgccgtccaggcacctcgattagttctcgagcttttggagtacgtcgtctttaggttggggggaggggttttatgcgatggagtttccccacactgagtgggtggagactgaagttaggccagcttggcacttgatgtaattctccttggaatttgccctttttgagtttggatcttggttcattctcaagcctcagacagtggttcaaagtttttttcttccatttcaggtgtcgtgagccatgggatccccgaagaaaaagcggaaagtcgaggcctccgcatctccaaaaaaaaaaagcaaggttgaagcatctggatccggtaccggaggaagtggcagctctggcggcagtggagggtctggtggcagcggaATGGAAGTTCAGCTGCAGGCAAGCGGTGGTGGTTTTGTTCAGCCTGGTGGTAGCCTGCGTCTGAGCTGTGCAGCCAGCGGTTTTACCTGGGATCATTACATCATGGGCTGGTTTCGCCAGGCACCGGGTAAAGAACGTGAATTTGTTAGCGCAATCAGCGAAAATGGTGATGCATGGAATTATTATGCCGATAGCGTGAAAGGTCGCTTTACCATTAGCCGTGATAATAGCAAAAATACCGTTTACCTGCAGATGAATAGTCTGCGTGCAGAAGATACCGCAACCTATTATTGTGCAATCGGTTTTGATGTTCCATCTGGTCGTTCTTGGCAGGGTTCTCATTTTTGGATGTATTGGGGTCAGGGCACCCAGGTTACCGTTAGCAGCTCGGACAGCGCCGGCAGCGCCGGCAGCGCCGGCAGCGGATCCCCTTCAGGGCAGATCAGCAACCAGGCCCTGGCTCTGGCCCCTAGCTCCGCTCCAGTGCTGGCCCAGACTATGGTGCCCTCTAGTGCTATGGTGCCTCTGGCCCAGCCACCTGCTCCAGCCCCTGTGCTGACCCCAGGACCACCCCAGTCACTGAGCGCTCCAGTGCCCAAGTCTACACAGGCCGGCGAGGGGACTCTGAGTGAAGCTCTGCTGCACCTGCAGTTCGACGCTGATGAGGACCTGGGAGCTCTGCTGGGGAACAGCACCGATCCCGGAGTGTTCACAGATCTGGCCTCCGTGGACAACTCTGAGTTTCAGCAGCTGCTGAATCAGGGCGTGTCCATGTCTCATAGTACAGCCGAACCAATGCTGATGGAGTACCCCGAAGCCATTACCCGGCTGGTGACCGGCAGCCAGCGGCCCCCCGACCCCGCTCCAACTCCCCTGGGAACCAGCGGCCTGCCTAATGGGCTGTCCGGAGATGAAGACTTCTCAAGCATCGCTGATATGGACTTTAGTGCCCTGCTGTCACAGATTTCCTCTAGTGGGCAGGGAGGAGGTGGAAGCGGCTTCAGCGTGGACACCAGTGCCCTGCTGGACCTGTTCAGCCCCTCGGTGACCGTGCCCGACATGAGCCTGCCTGACCTTGACAGCAGCCTGGCCAGTATCCAAGAGCTCCTGTCTCCCCAGGAGCCCCCCAGGCCTCCCGAGGCAGAGAACAGCAGCCCGGATTCAGGGAAGCAGCTGGTGCACTACACAGCGCAGCCGCTGTTCCTGCTGGACCCCGGCTCCGTGGACACCGGGAGCAACGACCTGCCGGTGCTGTTTGAGCTGGGAGAGGGCTCCTACTTCTCCGAAGGGGACGGCTTCGCCGAGGACCCCACCATCTCCCTGCTGACAGGCTCGGAGCCTCCCAAAGCCAAGGACCCCACTGTCTCCTctagagggcccgtttaaacccgctgatcagcctcgactgtgccttctagttgccagccatctgttgtttgcccctcccccgtgccttccttgaccctggaaggtgccactcccactgtcctttcctaataaaatgaggaaattgcatcgcattgtctgagtaggtgtcattctattctggggggtggggtggggcaggacagcaagggggaggattgggaagacaatagcaggcatgctggggatgcggtgggctctagaaagcaagtaaaacctctacaaatgtggtatggctgattatgatcctgcccgggtaggggaggcgcttttcccaaggcagtctggagcatgcgctttagcagccccgctgggcacttggcgctacacaagtggcctctggcctcgcacacattccacatccaccggtaggcgccaaccggctccgttctttggtggccccttcgcgccaccttctactcctcccctagtcaggaagttcccccccgccccgcagctcgcgtcgtgcaggacgtgacaaatggaagtagcacgtctcactagtctcgtgcagatggacagcaccgctgagcaatggaagcgggtaggcctttggggcagcggccaatagcagctttgctccttcgctttctgggctcagaggctgggaaggggtgggtccgggggcgggctcaggggcgggctcaggggcggggcgggcgcccgaaggtcctccggaggcccggcattctgcacgcttcaaaagcgcacgtctgccgcgctgttctcctcttcctcatctccgggcctttcgacctgcagcccaagcttaccATGACCGAGTACAAGCCCACGGTGCGCCTCGCCACCCGCGACGACGTCCCCAGGGCCGTACGCACCCTCGCCGCCGCGTTCGCCGACTACCCCGCCACGCGCCACACCGTCGATCCGGACCGCCACATCGAGCGGGTCACCGAGCTGCAAGAACTCTTCCTCACGCGCGTCGGGCTCGACATCGGCAAGGTGTGGGTCGCGGACGACGGCGCCGCGGTGGCGGTCTGGACCACGCCGGAGAGCGTCGAAGCGGGGGCGGTGTTCGCCGAGATCGGCCCGCGCATGGCCGAGTTGAGCGGTTCCCGGCTGGCCGCGCAGCAACAGATGGAAGGCCTCCTGGCGCCGCACCGGCCCAAGGAGCCCGCGTGGTTCCTGGCCACCGTCGGCGTCTCGCCCGACCACCAGGGCAAGGGTCTGGGCAGCGCCGTCGTGCTCCCCGGAGTGGAGGCGGCCGAGCGCGCCGGGGTGCCCGCCTTCCTGGAGACCTCCGCGCCCCGCAACCTCCCCTTCTACGAGCGGCTCGGCTTCACCGTCACCGCCGACGTCGAGGTGCCCGAAGGACCGCGCACCTGGTGCATGACCCGCAAGCCCGGTGCCTGAAGGCCTGTCGTGAAGCTTGGGGATCAATTCTCTAGAGCTCGCTGATCAGCCTCGACTGTGCCTTCTAGTTGCCAGCCATCTGTTGTTTGCCCCTCCCCCGTGCCTTCCTTGACCCTGGAAGGTGCCACTCCCACTGTCCTTTCCTAATAAAATGAGGAAATTGCATCGCATTGTCTGAGTAGGTGTCATTCTATTCTGGGGGGTGGGGTGGGGCAGGACAGCAAGGGG** |
| **pNX176: ITR-P_EF1α_-Gal4 DBD-FnBphP-polyA :: P_RL_-IFN-β-P2A-NanoLuc-bGH polyA :: P_mPGK_-ZeoR-P2A-EGFP-pA-ITR; P_RL,_ 5×UAS-P_hCMVmin_-kozak** |
| **gggaaagtgatgtcgtgtactggctccgcctttttcccgagggtgggggagaaccgtatataagtgcagtagtcgccgtgaacgttctttttcgcaacgggtttgccgccagaacacaggtaagtgccgtgtgtggttcccgcgggcctggcctctttacgggttatggcccttgcgtgccttgaattacttccacctggctgcagtacgtgattcttgatcccgagcttcgggttggaagtgggtgggagagttcgaggccttgcgcttaaggagccccttcgcctcgtgcttgagttgaggcctggcctgggcgctggggccgccgcgtgcgaatctggtggcaccttcgcgcctgtctcgctgctttcgataagtctctagccatttaaaatttttgatgacctgctgcgacgctttttttctggcaagatagtcttgtaaatgcgggccaagatctgcacactggtatttcggtttttggggccgcgggcggcgacggggcccgtgcgtcccagcgcacatgttcggcgaggcggggcctgcgagcgcggccaccgagaatcggacgggggtagtctcaagctggccggcctgctctggtgcctggcctcgcgccgccgtgtatcgccccgccctgggcggcaaggctggcccggtcggcaccagttgcgtgagcggaaagatggccgcttcccggccctgctgcagggagctcaaaatggaggacgcggcgctcgggagagcgggcgggtgagtcacccacacaaaggaaaagggcctttccgtcctcagccgtcgcttcatgtgactccacggagtaccgggcgccgtccaggcacctcgattagttctcgagcttttggagtacgtcgtctttaggttggggggaggggttttatgcgatggagtttccccacactgagtgggtggagactgaagttaggccagcttggcacttgatgtaattctccttggaatttgccctttttgagtttggatcttggttcattctcaagcctcagacagtggttcaaagtttttttcttccatttcaggtgtcgtgaGCCACCATGTGCGGCCGCAAGCTGCTGAGCAGCATCGAGCAGGCCTGCGACATCTGCAGGCTGAAGAAGCTGAAGTGTAGCAAGGAGAAGCCTAAGTGCGCCAAGTGCCTGAAGAACAACTGGGAGTGCAGGTATAGCCCCAAGACCAAGAGGAGCCCCCTGACCAGAGCCCACCTGACCGAAGTGGAGAGCAGGCTGGAGAGGCTGGAGCAGCTGTTCCTGCTGATCTTCCCCAGAGAAGACCTGGACATGATCCTGAAGATGGACAGCCTGCAGGATATCAAGGCCCTCCTGACAGGCCTGTTTGTCCAGGACAACGTCAACAAGGACGCCGTGACCGATAGACTGGCCTCTGTGGAGACAGACATGCCCCTGACCCTGAGGCAGCACAGGATCAGCGCCACCAGCTCCAGCGAGGAGTCCAGCAACAAGGGCCAGAGGCAGCTCACCGTGAGCGCCAGCGGAAGTGGCGGAGGAGGCGACGTCATGAGCGAGCTGCCCAGCAGATCCATCTCCCCTAGGGACCCTAGCCCCGGCGAGACCCCAGGAAGAGACCCTTCCACCCCTAGCACCGACGCCGGCGTGGGATACAGCGCCAGCCAGGATGCCCCCTCCTTTGGCGCCTACGATAGGGTGTACCCCATCAGGAGCCTGGTGAGCCTGGAGCCTCCCGCCACATCCGAGCCCTCCAGCAACAAGTCCAAGTCCCCTCTGAGCCCTACATCCGGCGCCAGACAGTTTTCCATCATCGACGGCCACACATGGACCAGGCTGAGAAGCGATTCCAGGGCCAACTCCACCGACTACTCCGGCGGCACAGGCCTGTCCCCCGAGTCTAGCGAGGCCCCTTCCAGCCAGAGGATGTCCGACTCCAGCTCCGCCAGGCCTCCCTCCAACACCACAGGCCTGAGGAGAGGCGATGATCACACCACCTTTACCCCTAGCAGCGAGGACAGCCACCCTCAGGTGCAGGAGCCCTACGAGCTGATGACCACAAGGTTTAGACACGTGGTGACCGATGACGGCCACGCCGTGATCACCGGCAGGACCGTGGATAGCTTTAAGGCcATGAGCAGAGATCCCCTGCCTTTCTTCCCACCACTGTACCTCGGAGGCCCTGAGATCACCACCGAGAACTGCGAGAGAGAGCCCATTCACATCCCCGGCTCTATTCAGCCTCATGGCGCTCTGCTGACAGCCGATGGACATTCTGGCGAGGTGCTGCAGATGAGCCTGAATGCCGCCACCTTCCTGGGCCAAGAACCTACCGTTCTGAGAGGCCAGACACTGGCTGCACTGCTGCCTGAACAATGGCCTGCTCTGCAAGCTGCTCTGCCTCCTGGATGTCCTGACGCTCTGCAGTACAGAGCCACACTGGATTGGCCTGCCGCCGGACATCTGTCTCTGACAGTGCACAGAGTGGGCGAGCTGCTGATCCTGGAATTCGAGCCTACAGAGGCCTGGGACTCTACAGGACCTCACGCTCTGAGAAACGCCATGTTCGCCCTGGAAAGCGCCCCTAATCTGAGAGCCCTGGCCGAAGTGGCTACCCAGACAGTCAGAGAGCTGACCGGCTTCGACAGAGTGATGCTGTACAAGTTCGCCCCTGACGCCACCGGCGAAGTGATTGCCGAAGCCAGAAGAGAAGGCCTGCACGCCTTTCTGGGCCACAGATTTCCAGCCAGCGACATCCCTGCTCAGGCTAGAGCCCTGTACACCCGGCATCTGCTGAGACTGACCGCCGATACAAGAGCCGCTGCTGTGCCACTGGACCCCGTTCTGAACCCTCAGACAAACGCCCCTACACCTCTTGGCGGAGCTGTGCTGAGAGCCACCTCTCCTATGCACATGCAGTACCTGCGGAACATGGGCGTGGGAAGCAGCCTGTCTGTGTCTGTGGTTGTTGGCGGACAGCTGTGGGGACTGATCGCCTGTCATCACCAGACACCTTACGTGCTGCCTCCAGACCTGCGGACAACCCTGGAATATCTGGGCAGACTGCTGAGCCTGCAGGTCCAAGTGAAAGAGGCTGCTGACGTCGCCGCCTTCAGACAGAGCCTGAGAGAACACCATGCCAGAGTGGCACTGGCCGCTGCTCATTCTCTGAGCCCTCACGATACCCTGAGCGACCCTGCTCTGGATCTGCTGGGACTTATGAGAGCCGGCGGACTGATCCTGAGATTTGAAGGCAGATGGCAGACCCTGGGAGAAGTGCCTCCTGCTCCTGCTGTTGATGCTCTGCTGGCCTGGCTCGAAACACAACCTGGTGCTCTGGTGCAGACAGATGCCCTGGGACAACTTTGGCCTGCTGGCGCTGATCTGGCTCCTTCTGCTGCTGGACTGCTCGCCATCTCTGTTGGAGAAGGCTGGAGCGAGTGTCTCGTGTGGCTCAGACCTGAGCTGAGGCTGGAAGTTGCTTGGGGCGGAGCTACACCCGATCAGGCCAAGGATGATCTGGGCCCCAGACACAGCTTCGACACCTACCTGGAAGAGAAGCGGGGCTATGCCGAACCTTGGCACCCTGGCGAAATTGAGGAAGCCCAGGACCTGAGGGACACACTGACAGGTGCTCTTcTCGAGcTCGAGAATAAAATATCTTTATTTTCATTACATCTGTGTGTTGGTTTTTTGTGTGAATCGATAGTACTAACATACGCTCTCCATCAAAACAAAACGAAACAAAACAAACTAGCAAAATAGGCTGTCCCCAGTGCAAGTGCAGGTGCCAGAACATTTCTCTATCGATCGATAGGTACCCGGAGTACTGTCCTCCGAGCGGAGTACTGTCCTCCGAGCGGAGTACTGTCCTCCGAGCGGAGTACTGTCCTCCGAGCGGAGTACTGTCCTCCGAGCctgcaggTCGAGCTCGGTACCCGGGTCGAGTAGGCGTGTACGGTGGGAGGCCTATATAAGCAGAGCTCGTTTAGTGAACCGTCAGATCGCCTGGAGACGCCATCCACGCTGTTTTGACCTCCATAGAAGACACCGGGACCGATCCAGCCTCCGCGgAATTCGAGCTCGCCCGGGGATCCGCCACCATGAACAACAGGTGGATCCTCCACGCTGCGTTCCTGCTGTGCTTCTCCACCACAGCCCTCTCCATCAACTATAAGCAGCTCCAGCTCCAAGAAAGGACGAACATTCGGAAATGTCAGGAGCTCCTGGAGCAGCTGAATGGAAAGATCAACCTCACCTACAGGGCGGACTTCAAGATCCCTATGGAGATGACGGAGAAGATGCAGAAGAGTTACACTGCCTTTGCCATCCAAGAGATGCTCCAGAATGTCTTTCTTGTCTTCAGAAACAATTTCTCCAGCACTGGGTGGAATGAGACTATTGTTGTACGTCTCCTGGATGAACTCCACCAGCAGACAGTGTTTCTGAAGACAGTACTAGAGGAAAAGCAAGAGGAAAGATTGACGTGGGAGATGTCCTCAACTGCTCTCCACTTGAAGAGCTATTACTGGAGGGTGCAAAGGTACCTTAAACTCATGAAGTACAACAGCTACGCCTGGATGGTGGTCCGAGCAGAGATCTTCAGGAACTTTCTCATCATTCGAAGACTTACCAGAAACTTCCAAAACGGGAGCGGCGCCACAAACTTTTCCCTCCTGAAGCAGGCTGGAGATGTGGAGGAGAATCCCGGACCTAGCGGAATGACTAGTGAGACAGACACACTCCTGCTATGGGTACTGCTGCTCTGGGTTCCAGGTTCCACTGGTGACGCTAGTggtggttctggtATGGTCTTCACACTCGAAGATTTCGTTGGGGACTGGCGACAGACAGCCGGCTACAACCTGGACCAAGTCCTTGAACAGGGAGGTGTGTCCAGTTTGTTTCAGAATCTCGGGGTGTCCGTAACTCCGATCCAAAGGATTGTCCTGAGCGGTGAAAATGGGCTGAAGATCGACATCCATGTCATCATCCCGTATGAAGGTCTGAGCGGCGACCAAATGGGCCAGATCGAAAAAATTTTTAAGGTGGTGTACCCTGTGGATGATCATCACTTTAAGGTGATCCTGCACTATGGCACACTGGTAATCGACGGGGTTACGCCGAACATGATCGACTATTTCGGACGGCCGTATGAAGGCATCGCCGTGTTCGACGGCAAAAAGATCACTGTAACAGGGACCCTGTGGAACGGCAACAAAATTATCGACGAGCGCCTGATCAACCCCGACGGCTCCCTGCTGTTCCGAGTAACCATCAACGGAGTGACCGGCTGGCGGCTGTGCGAACGCATTCTGGCGgCTAGTTCCGGTTGTAAGCCTTGCATATGTACAGTCCCAGAAGTATCATCTGTCTTCATCTTCCCCCCAAAGCCCAAGGATGTGCTCACCATTACTCTGACTCCTAAGGTCACGTGTGTTGTGGTAGACATCAGCAAGGATGATCCCGAGGTCCAGTTCAGCTGGTTTGTAGATGATGTGGAGGTGCACACAGCTCAGACGCAACCCCGGGAGGAGCAGTTCAACAGCACTTTCCGCTCAGTCAGTGAACTTCCCATCATGCACCAGGACTGGCTCAATGGCAAGGAGTTCAAATGCAGGGTCAACAGTGCAGCTTTCCCTGCCCCCATCGAGAAAACCATCTCCAAAACCAAAGGCAGACCGAAGGCTCCACAGGTGTACACCATTCCACCTCCCAAGGAGCAGATGGCCAAGGATAAAGTCAGTCTGACCTGCATGATAACAGACTTCTTCCCTGAAGACATTACTGTGGAGTGGCAGTGGAATGGGCAGCCAGCGGAGAACTACAAGAACACTCAGCCCATCATGGACACAGATGGCTCTTACTTCGTCTACAGCAAGCTCAATGTGCAGAAGAGCAACTGGGAGGCAGGAAATACTTTCACCTGCTCTGTGTTACATGAGGGCCTGCACAACCACCATACTGAGAAGAGCCTCTCCCACTCTCCTGGTAAAgCTAGCGGATCCACCGGTgTCTAGTAATCTAGAcagacatgataagatacattgatgagtttggacaaaccacaactagaatgcagtgaaaaaaatgctttatttgtgaaatttgtgatgctattgctttatttgtaaccattataagctgcaataaacaagttaacaacaacaattgcattcattttatgtttcaggttcagggggaggtgtgggaggttttttgaaagcaagtaaaacctctacaaatgtggtatggctgattatgatcctgcccgggtaggggaggcgcttttcccaaggcagtctggagcatgcgctttagcagccccgctgggcacttggcgctacacaagtggcctctggcctcgcacacattccacatccaccggtaggcgccaaccggctccgttctttggtggccccttcgcgccaccttctactcctcccctagtcaggaagttcccccccgccccgcagctcgcgtcgtgcaggacgtgacaaatggaagtagcacgtctcactagtctcgtgcagatggacagcaccgctgagcaatggaagcgggtaggcctttggggcagcggccaatagcagctttgctccttcgctttctgggctcagaggctgggaaggggtgggtccgggggcgggctcaggggcgggctcaggggcggggcgggcgcccgaaggtcctccggaggcccggcattctgcacgcttcaaaagcgcacgtctgccgcgcatggccaagttgaccagtgccgttccggtgctcaccgcgcgcgacgtcgccggagcggtcgagttctggaccgaccggctcgggttctcccgggacttcgtggaggacgacttcgccggtgtggtccgggacgacgtgaccctgttcatcagcgcggtccaggaccaggtggtgccggacaacaccctggcctgggtgtgggtgcgcggcctggacgagctgtacgccgagtggtcggaggtcgtgtccacgaacttccgggacgcctccgggccggccatgaccgagatcggcgagcagccgtgggggcgggagttcgccctgcgcgacccggccggcaactgcgtgcacttcgtggccgaggagcaggacggaagcggagctactaacttcagcctgctgaagcaggctggagacgtggaggagaaccctggaccttccggagtgagcaagggcgaggagctgttcaccggggtggtgcccatcctggtcgagctggacggcgacgtaaacggccacaagttcagcgtgtccggcgagggcgagggcgatgccacctacggcaagctgaccctgaagttcatctgcaccaccggcaagctgcccgtgccctggcccaccctcgtgaccaccctgacctacggcgtgcagtgcttcagccgctaccccgaccacatgaagcagcacgacttcttcaagtccgccatgcccgaaggctacgtccaggagcgcaccatcttcttcaaggacgacggcaactacaagacccgcgccgaggtgaagttcgagggcgacaccctggtgaaccgcatcgagctgaagggcatcgacttcaaggaggacggcaacatcctggggcacaagctggagtacaactacaacagccacaacgtctatatcatggccgacaagcagaagaacggcatcaaggtgaacttcaagatccgccacaacatcgaggacggcagcgtgcagctcgccgaccactaccagcagaacacccccatcggcgacggccccgtgctgctgcccgacaaccactacctgagcacccagtccgccctgagcaaagaccccaacgagaagcgcgatcacatggtcctgctggagttcgtgaccgccgccgggatcactctcggcatggacgagctgtacaagtaaTCTAGAGCTCGCTGATCAGCCTcagacatgataagatacattgatgagtttggacaaaccacaactagaatgcagtgaaaaaaatgctttatttgtgaaatttgtgatgctattgctttatttgtaaccattataagctgcaataaacaagttaacaacaacaattgcattcattttatgtttcaggttcagggggaggtgtgggaggtttttt** |
